# Enhanced Th1 Cellular Immunity Induced by an RSV-F mRNA Vaccine Rationally Designed Using NLP Algorithms

**DOI:** 10.3390/vaccines14040356

**Published:** 2026-04-16

**Authors:** Zhi-Wu Xia, Qi Tang, Jun-Jie Pan, Jing Liu, Lan-Xin Jia, Guo-Mei Zhang, Man-Ni Xie, Jia-Hao Zheng, Chuan-Shuo Lv, Lei Zhang, Yan-Hong Shi, Liang He, Min Luo, Jun-Long Zhao

**Affiliations:** 1 College of Veterinary Medicine, Huazhong Agricultural University, Wuhan 430070, China; 2 National Engineering Technology Research Center for Combined Vaccines, Wuhan 430207, China; 3 Wuhan Institute of Biological Products Co., Ltd., Wuhan 430207, China; 4 National Key Laboratory of Agricultural Microbiology, College of Veterinary Medicine Huazhong Agricultural University, Wuhan 430070, China

**Keywords:** respiratory syncytial virus, mRNA vaccine, immunogenicity evaluation, T-cell immunity, memory T cells

## Abstract

**Background:** Respiratory syncytial virus (RSV) is a leading cause of severe lower respiratory tract infections in infants, seniors, and immunocompromised individuals, contributing substantially to the global disease burden. Given the limited preventive options available, developing an effective and safe vaccine remains a public health priority. **Methods:** An mRNA vaccine encoding the RSV PreF protein was designed and prepared. Antigen properties were evaluated in silico, and the coding sequence was optimized using NLP algorithms. The stability and translational efficiency of the mRNA constructs were verified through in vitro and in vivo assays, followed by immunogenicity evaluation of the formulated mRNA vaccines in a BALB/c mouse model. **Results:** The optimized mRNA showed predicted improvements in structural stability and a lower free energy state, which were associated with increased translational efficacy in vitro. Correct antigen conformation and retention of key epitopes were confirmed by intracellular staining followed by flow cytometry. A balanced Th1-biased immune response was induced in mice, characterized by high levels of neutralizing antibodies and antigen-specific T-cell immunity, along with enhanced memory T-cell proliferation and differentiation, indicating long-term immunological memory. **Conclusions:** A novel RSV PreF mRNA vaccine was successfully developed via optimization of protein structure and mRNA sequence. Superior immunogenicity was demonstrated in the BALB/c mouse model, together with promising potential in terms of vaccine safety and immunological persistence. These findings represent a promising step forward in the pursuit of an effective RSV vaccine and suggest the potential of the developed mRNA vaccine to induce substantial immune responses that may correlate with protection in future challenge studies.

## 1. Introduction

Respiratory syncytial virus (RSV) is a globally prevalent pathogen that causes substantial respiratory disease each year [1,2,3]. Although most infections cause only mild cold-like symptoms, severe illness occurs in 3–7% of susceptible individuals, particularly infants and the elderly [4,5]. Although precise global estimates for older adults are lacking, a substantial burden has been documented in the United States, where RSV accounts for approximately 160,000 hospitalizations and 10,000 deaths annually among individuals aged ≥65 years [3,6]. China, too, is recognized as a country with high RSV prevalence. In China, RSV accounts for approximately 3.5 million cases of acute lower respiratory tract infections (ALRIs) and 620,000–950,000 subsequent hospitalizations each year. These figures represent >10% and 17–24% of the global burden, respectively, ranking China second worldwide after India [7]. This situation has created a strong demand for RSV prevention, both in clinical settings and across society [8]. To address this need, the development and deployment of an effective RSV vaccine is urgently required to reduce morbidity and mortality [9,10].

Currently, more than 60 RSV vaccine candidates are under development with various technical platforms, including mainly subunit protein vaccines, mRNA vaccines, viral vector vaccines, and live attenuated vaccines [11]. Among these strategies, subunit and mRNA vaccines have demonstrated favorable safety profiles and high immunogenicity in clinical applications [12,13,14]. Unfortunately, a number of common concerns have been raised in applications [15]. First, limitations in immunogenicity and long-term protective efficacy have been observed. For instance, Arexvy showed a continuous decrease in efficacy during the second and third epidemic seasons (56.1% and 48%, respectively) [16]. A similar trend was also demonstrated with Abrysvo, the other approved subunit vaccine [17,18]. For the mRNA vaccine, the mRESVIA achieved only a 66.6% seroresponse rate against RSV-B in high-risk adults aged 50–59 years. On the other hand, safety concerns have also been highlighted [13]. The FDA has reported an association between Guillain–Barré syndrome (GBS), a rare neurological disorder, and vaccination with Arexvy or Abrysvo [19]. Furthermore, the lack of approved vaccines for infants and the potential risk of vaccine-enhanced respiratory disease (VERD) remain key challenges [20,21,22]. In summary, more efforts should be made to develop new RSV vaccines to facilitate both efficacy and safety.

Essentially, antigen design is always among the fundamental questions in the development of vaccines, including RSV vaccines. As demonstrated in a panel of investigations, the fusion protein (F), the key structural protein of RSV, has been recognized as a promising target for vaccine design [23]. There are two natural conformations of this protein: one is the metastable state of prefusion (PreF), and the other is stable postfusion (PostF) [24]. Notably, an irreversible transition from PreF to PostF was characterized during membrane fusion between the virus and host cells, in which a comprehensive change in epitope exposure consequently occurred [24]. Of particular importance, the site Φ epitope, the principal inducer of neutralizing antibodies, was maintained exclusively in PreF but never in the PostF conformation [25]. Similarly, the elicitation of neutralizing antibodies was robust to PreF but incompetent with PostF conformation, while substantial amounts of nonneutralizing antibodies were generated simultaneously [26]. The reshaped pattern of antibody production was subsequently correlated with a distinct risk of VERD [22]. Owing to its ability to induce neutralizing antibodies, primary attention has been given to stabilizing and utilizing the PreF conformation. For this purpose, structural biology techniques, such as targeted mutagenesis and disulfide bond engineering, are commonly employed thereafter, and some variants of the F protein that are useful for vaccine development have been generated and evaluated [26,27,28]. This approach of conformational stabilization is a cornerstone of modern structure-based vaccine design for emerging pathogens [29].

On the other hand, the choice of vaccine platform is important for addressing issues associated with RSV subunit vaccines (Arexvy and Abrysvo), such as potential GBS risk, insufficient immunogenicity and limited durability of protection [13,21]. For instance, mRNA vaccines offer the following distinct advantages: (1) In situ antigen synthesis within host cells avoids complex exogenous recombinant production and minimizes epitope loss because of conformational instability; and (2) effective activation of the MHC class I antigen presentation pathway stimulates a robust Th1-biased cellular immune response, enhancing clearance of virus-infected cells [30,31]. Additional benefits include strong immunogenicity, favorable safety, short development cycles, and high design flexibility, making the platform suitable for emerging pathogens and conformation-sensitive antigens such as RSV PreF [32]. Recent successes with mRNA vaccines against influenza, mpox, and rabies viruses highlight advantages in universality, rapid response capability to new outbreaks or variants, and reduced vaccination schedules for fast protection [33,34,35].

Nevertheless, key challenges remain, particularly in mRNA sequence design for stability and antigenicity. While strategies such as codon optimization, nucleoside modification, and structural refinement increase expression, stability, and immunogenicity, the optimization process remains complicated [36]. Fortunately, advances in artificial intelligence (AI) are illuminating this field, aiding in sequence design and optimization through the use of algorithmic models such as mRNArchitect, mRNAdesigner, and LinearDesign [37,38]. Notably, linear design, a natural language processing (NLP) model developed by Baidu Research, enabled the design of a COVID-19 mRNA vaccine in only 11 min while simultaneously optimizing mRNA stability and codon usage efficiency, demonstrating its considerable industrial potential [38].

In the current work, a linear design algorithm was adopted to design a novel RSV mRNA vaccine on the basis of the engineering-stabilized PreF conformation of the F protein. Both computational simulations and mouse model experiments were subsequently carried out to validate the global performance of our vaccine candidate. The physicochemical, structural and immunologic properties of the designed antigens were evaluated, and subsequently, an increase in protein translation efficiency was achieved with the MOF-mRNA (Modified and Optimized F-mRNA) vaccine after sequence optimization. An enhanced display of site Φ and a tendency toward preF conformation were subsequently confirmed. Stronger immunogenic responses, including RSV-specific humoral and Th1-biased cellular immunity, were triggered. In addition, the vaccine activated the proliferation and differentiation of CD4^+^ Tm cells, suggesting the induction of long-term immunological memory (See Appendix A). These findings highlight the potential application of this design.

## 2. Materials and Methods

### 2.1. Evaluation of Antigenic Proteins in Terms of Physicochemical and Immune Properties

The physicochemical properties of the antigenic proteins, including the isoelectric point (pI), molecular weight, half-life, stability and other essential descriptors, were computed with the ExPASy ProtParam Tool [39]. An instability index less than 40 indicates a stable molecule. Solubility was predicted with SOLpro and Protein-Sol [40], and a threshold > 0.5 was applied to confirm that the selected molecules remained soluble and functional under physiological conditions.

Antigenicity was evaluated with the VaxiJen v2.0 and ANTIGENpro servers [41], while allergenicity was assessed using AllerTOP v2.0 [42]. For epitope prediction, Bepipred 2.0 was used for B-cell epitopes, whereas NetMHC 4.0 was used for CTLs (MHC I), and NetMHC class II 2.3v was used for HTLs (MHC II). The lengths of the epitopes were defined as 8, 9, 10, and 11 aa for MHC I and 15 aa for MHC II.

### 2.2. Prediction and Evaluation of Protein Tertiary Structure

Tertiary structures of the antigenic proteins were modeled and evaluated with Swiss-Prot. The GDT-HA, RMSD, MolProbity score, Clash score, poor rotamers and Rama score were scored for each structural model to comprehensively determine the models of choice. The selected models were visualized in PyMOL 2.5. The overall quality was evaluated with the online platforms ProSA-web and PROCHECK on the basis of Z scores and Ramachandran plots, respectively. Assessment was also conducted in fractions to determine the local energy level by ProSA-web and rationality by ERRAT2.

### 2.3. Docking and Analysis of Protein-Immune Receptor Complexes

Docking of the protein against pattern-recognition receptors was performed with Hawkdock to prompt the potential areas of interaction in either vaccines or receptors and subsequently generate models of complexes with Haddock [43]. The resulting models were visualized in PyMOL. Binding energy and interface properties were interpreted using Prodigy and PDBePISA [44].

### 2.4. Designing, Optimization and Evaluation of mRNA Sequences

The mRNA coding sequences were generated and optimized on the website https://rna.baidu.com/cn/lineardesign (accessed on 15 March 2023). The secondary structure and energy characteristics of mRNA molecules were predicted using RNAfold [45]. Minimum free energy (MFE) and Gibbs free energy (ΔG) calculated by RNAfold were used to determine the quality of mRNA structures.

### 2.5. Preparation and Characterization of mRNA Vaccine Candidates

The plasmid template for in vitro transcription (IVT) was constructed on the basis of the pBluescript II SK(+) vector, which contains the following essential elements: a 5′ UTR, an antigen-coding sequence, a 3′ UTR, and a poly(A) tail. Nucleoside-modified mRNA was synthesized using a T7 High Yield RNA Transcription Kit (N1-Me-Pseudo UTP) (Vazyme, Cat. No. DD4202, Vazyme Biotech Co., Ltd., Nanjing, China) and a Cap 1 Capping System (Novoprotein, Cat. No. M082), followed by purification with lithium chloride (Thermo, Cat. No. AM9480, Thermo Fisher Scientific Inc., Waltham, MA, USA). mRNA integrity was verified via capillary electrophoresis and agarose gel electrophoresis, and aliquots were stored at −80 °C until use. For LNP formulation, mRNA was diluted to 100–200 ng/μL in sodium citrate buffer (pH 4.0) and stored at −80 °C. Lipids (DSPC, DMG-PEG2000, SM-102, and CHO-HP) were dissolved in anhydrous ethanol at optimized molar ratios, mixed at equal volumes, and combined with mRNA via microfluidics to form mRNA-LNPs. After formulation, the LNPs were concentrated through ultrafiltration and sterilized by 0.22-μm filtration. The mRNA concentration and encapsulation efficiency were quantified using a RiboGreen RNA Assay Kit (Invitrogen, Cat. No. R11490, Thermo Fisher Scientific Inc., Eugene, OR, USA). The particle size, polydispersity index (PDI), and zeta potential were measured by dynamic light scattering (Malvern Zetasizer, Malvern Panalytical Ltd., Malvern, UK).

### 2.6. Validation of the mRNA Vaccine

HEK-293T cells were seeded in 24-well plates and transfected at 80% confluency with 2.5 μg of mRNA/well using Lipo8000™ Transfection Reagent (Boyetime, Cat. No. C0533, Beijing Boyetime Biotechnology Co., Ltd., Beijing, China). After 24 h, the cells were lysed and subjected to Western blotting to verify F protein expression.

For flow cytometry analysis, a single-cell suspension was enzymatically collected from HEK-293T cells post transfection. Cells were stained with two panels of antibodies, one containing FITC-3C12 (Novoprotein, Cat. No. DA101FDL, Novoprotein Scientific Inc., Summit, NJ, USA) and PE-4D5 (Novoprotein, Cat. No. DA102BDL), and the other containing FITC-7H11 (Novoprotein, Cat. No. DA109FDL) and PE-4D5. All antibodies and isotype controls were used at a final concentration of 5 μg/mL. Staining was performed in the dark for 10 min at room temperature with viability dye and then for 30 min on ice with antibodies.

### 2.7. Mouse Immunization and Sample Collection

Female BALB/c mice (6–8 weeks old, SPF) were obtained from Wuhan Institute of Biological Products Co., Ltd. (Wuhan, China). The animals were housed under specific pathogen-free (SPF) conditions with 12 h light/dark cycles and ad libitum access to food and water. Four groups of 11 mice each were randomly established: Group 1 was the PBS control group, and Groups 2, 3, and 4 were different vaccine groups. All BALB/c mice completed the immunization schedule and were included in the final analysis, with no exclusions. Mice from the same shipment were distributed evenly across groups, cage positions were randomized, and all procedures were performed at a consistent time of day to control for circadian variation. The animals were immunized with distinct vaccine candidates or with PBS as a negative control. Intramuscular immunizations were conducted twice, with 10 μg mRNA per dose and an interval of 21 days. Subsets (*n* = 5–6) were randomly selected from the original cohort of 11 mice per group for blood and spleen collection at day 14, and spleen collection only at day 120 post-boost.

### 2.8. ELISA

To detect PreF-specific total IgG, PreF protein was diluted to 2 μg/mL in coating buffer (100 μL/well) and incubated in 96-well plates at 2–8 °C overnight. After blocking with 2% BSA (200 μL/well, 37 °C, 60 min), serum samples were initially diluted 200-fold, followed by 2-fold serial dilutions (100 μL/well, room temperature, 60 min). The plates were subsequently washed with PBST, incubated with HRP-conjugated goat anti-mouse IgG (1:5000, 100 μL/well, room temperature, 60 min), developed with TMB substrate (100 μL/well, room temperature, 10 min), and stopped with 2 M sulfuric acid (50 μL/well). Optical density (OD) was measured at 450 nm using a Multiskan™ FC microplate reader. The endpoint titer was defined as the highest serum dilution with an OD value exceeding 2.1-fold that of the negative control. Nonspecific IgG1 and IgG2a levels were measured using commercial ELISA kits (Sangon Biotech: IgG1 Cat. D721096; IgG2a Cat. D721097, Sangon Biotech Co., Ltd., Shanghai, China).

### 2.9. Neutralization Assay

Hep-2 cells were seeded in 96-well plates and used when they reached 95% confluency. Heat-inactivated mouse serum was diluted with DMEM supplemented with 2% FBS in a 96-well plate as follows: the initial dilution was 8-fold in column #1, and then the serial dilutions were kept 2-fold across columns #2-#11, while column #12 was used as a negative control. RSV long-strain virus (200 CCID50/100 μL, 50 μL/well) was added, mixed, and incubated with diluted mouse serum at 37 °C for 2 h. The serum-virus mixtures were then transferred to Hep-2 cell monolayers and cultured at 37 °C and 5% CO_2_ for 5~7 days. Cytopathic effects were observed, and RSV-specific neutralizing antibody titers were calculated using the Reed–Muench method.

### 2.10. Flow Cytometric Analysis of T Lymphocytes

To analyze splenic T lymphocyte subsets, the spleens from immunized mice were homogenized to obtain single-cell suspensions. Lymphocytes were isolated using Mouse 1× Lymphocyte Separation Medium (Dakewe, Cat. #7211011, Dakewe Biotech Co., Ltd., Shenzhen, China), washed twice with PBS, and stimulated with a Respiratory Syncytial Virus Fusion Protein Peptide pool (Vazyme, Cat. #DD9123, 2 μg/mL, Vazyme Biotech Co., Ltd., Nanjing, China) plus CD28 (BD Biosciences, Cat. #553294, BD Biosciences, San Jose, CA, USA) for 18 h. Brefeldin A (BFA) was added and maintained for the final 5 h. First, the cells were stained with a Zombie Aqua Fixable Viability Kit (Biolegend, Cat. #423102, 10 min, RT, BioLegend, Inc., San Diego, CA, USA), followed by APC/Cyanine7 anti-mouse CD3 (Biolegend, Cat. #100330), PerCP anti-mouse CD4 (Biolegend, Cat. #100434), and FITC anti-mouse CD8 (Biolegend, Cat. #100706) for 15–30 min at RT. After fixation (Biolegend Fixation Buffer, Cat. #420801) and permeabilization (Biolegend Intracellular Staining Permeabilization Wash Buffer, Cat. #421002), intracellular cytokines were stained with PE-conjugated anti-mouse IL-2 (Biolegend, Cat. #503808), BV605-conjugated anti-mouse IL-4 (Biolegend, Cat. #504126), APC-conjugated anti-mouse TNF-α (Biolegend, Cat. #506308), and BV421-conjugated anti-mouse IFN-γ (Biolegend, Cat. #505830) for 15 min at RT.

Memory T cells were also analyzed in isolated splenocytes after staining with BB700 anti-mouse CD3 (BioLegend, Cat. #566494), FITC anti-mouse CD4 (BioLegend, Cat. #100516), APC anti-mouse CD8 (BioLegend, Cat. #100706), PE/Cy7 anti-mouse CD44 (BioLegend, Cat. #103049), and BV605 anti-mouse CD62L (BioLegend, Cat. #104428) antibodies for 15–30 min at RT. CD44^+^CD62L^+^ and CD44^+^CD62L-subpopulations were quantified as central and effector memory T cells, respectively.

All the samples were analyzed on a Beckman CytoflexS (Beckman Coulter Inc., Brea, CA, USA). Fluorechrome compensation was set up with single-stained controls.

### 2.11. Statistical Analysis

In vitro experiments were independently repeated in triplicate. All replication attempts were successful. Animal experiments were completed once. The sample size of 11 mice per group was determined based on prior RSV vaccine studies with similar experimental designs, ensuring adequate statistical power (>80%) to detect biologically meaningful differences in antibody responses and cellular immunity. All the data were analyzed using GraphPad Prism 9.5. Antibody titers were log-transformed before analysis. Normality was assessed by the Shapiro–Wilk test, and homogeneity of variance was evaluated by Brown–Forsythe or Bartlett’s tests. On the basis of these results, appropriate statistical tests were selected: if both assumptions were met, ordinary one-way ANOVA was used, followed by Tukey’s multiple comparisons test; if the data were normally distributed but variances were unequal, Welch’s ANOVA was applied, followed by Games-Howell or Dunnett’s T3 tests; if the normality assumption was violated, the Kruskal–Wallis test was performed, followed by Dunn’s multiple comparisons test. Statistical significance was considered when *p* < 0.05. Exact *p*-values for all statistical comparisons are provided in Supplementary Materials S3.

## 3. Results

### 3.1. MF-F Mutant Was Suggested as a Promising Immunogen via In Silico Analysis

**Figure 1:**
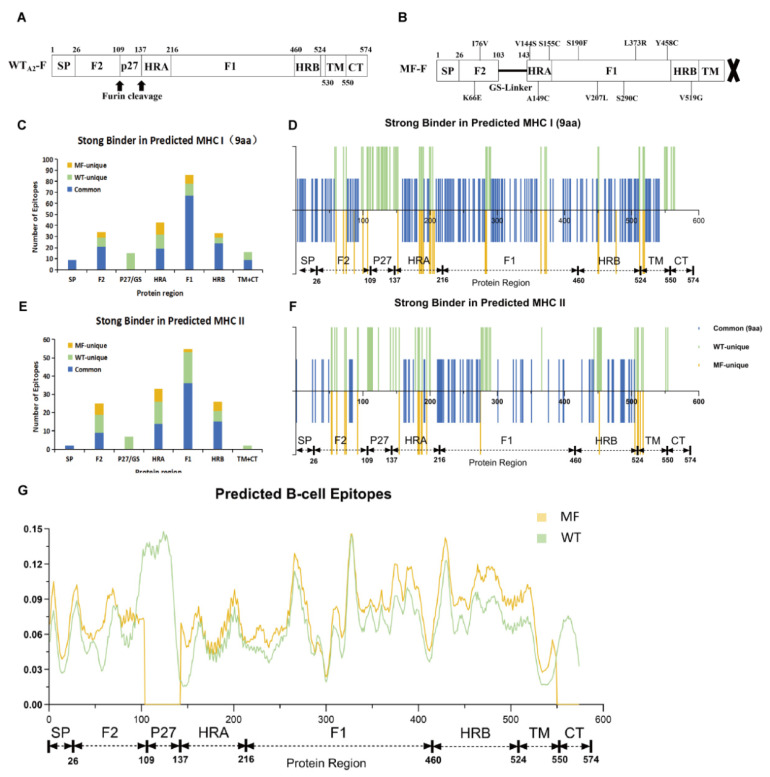
Design of F protein variant and prediction of T/B cell epitopes. Structural schematics of (**A**) wildtype F protein of RSV A2 strain and (**B**) its variant MF. (**C**–**F**) Distribution of T cell epitopes predicted in WT and MF proteins, including (**C**,**D**) 9aa strong binding epitopes of class I and (**E**,**F**) T cell class II epitopes. (**C**,**E**) In each domain, number of epitopes was summarized, and also, (**D**,**F**) distribution of epitopes was aligned between WT and MF proteins. The epitopes common for both proteins were indicated in blue, epitopes unique to WT protein in light green, and epitopes unique to MF protein in orange-yellow. (**G**) Alignment of the scores of antigenicity of WT and MF proteins predicted by Bepipred3.0 tool. Structural schematics of (**A**) wildtype F protein of RSV A2 strain and (**B**) its variant MF (A cross denotes the deletion of the CT region.).

Bioinformatics analyses were first employed to comparatively predict the physicochemical properties, immunological characteristics, and T/B cell epitopes of the designed F protein. Preliminary in silico assessment of basic properties and immunogenicity was conducted to guide subsequent studies. The structure of the WT-F (Wild-Type F protein) of the RSV A2 strain (Gen Bank: X02221.1) was used as a control vaccine (Figure 1A). To generate the MF (Modified F) mutant, the p27 peptide, spanning from aa 103~aa 143 between F2 and HRA, was replaced with a GS linker first. The C-terminal fragment was then removed after aa 550. Additional point mutations (e.g., K66E, I76V, V144S, A149C, L373R, Y458C and V519G) were introduced in addition to S155C, S190F, V207L, and S290C, and the mutations were in the DS-Cav1 variant, resulting in a total of 11 point mutations (Figure 1B). With these modifications, the trimer of the F protein is then formed via the expected disulfide bonds between monomers and thereby stabilized in the PreFusion conformation.

Despite the structural changes, favorable physicochemical and immunological properties were favorable for maintaining the MF-F protein at similar levels as those of the WT-F protein (Table 1). In addition to slight decreases in molecular mass from 63 kDa to 56 kDa and the pI from 9.17 to 8.28, both proteins were stable, with instability indices < 40, aliphatic indices > 100 (indicating thermostability), and GRAVY values near zero (moderate hydrophilicity). Both proteins were predicted to be nonallergenic and highly antigenic (antigenicity score > 0.88).

With the prediction of T-cell epitopes and comparisons between the WT-F and MF-F proteins (Figure 1C–F, Figure S1 and Figure S2 and Table S1), significant alterations were revealed in both the regional summary (Figure 1C,E and Figure S2A,C,E) and the sequential distribution (Figure 1D,F and Figure S2B,D,F). Owing to p27 replacement, C-terminal deletion and a panel of point mutations, a panel of epitopes were found uniquely in WT-F and absent in MF-F (green), while some others were found only in MF (yellow). Obviously, the majority of WT unique epitopes (green) were located mainly in the regions of replacement or deletion, p27 and CT. Other unique mutations, whether in the WT (green) or MF (yellow), were associated with the point mutations introduced, which clearly showed a sequential distribution pattern. However, the epitopes common to WT-F and MF-F (blue) still remained dominant and were enriched mainly in conserved domains such as F1, F2 and HRA. Interestingly, a dominant portion of unique MF epitopes were concentrated within the HRA and HRB, and these two regions were relatively smaller but tightly correlated with the induction of neutralizing antibodies and T-cell responses upon viral invasion. On the other hand, the overall prediction of the B-cell epitope revealed higher scores for MF-F in all structural regions except P27 and CT than for WT-F (Figure 1G), which suggested a greater potential to induce humoral immunity.

### 3.2. Robust Interactions Between Vaccine Candidates and Immune Receptors Were Predicted with Molecular Docking

Subsequently, the interaction efficacy between the candidate vaccine and immune receptors was further investigated by molecular docking. First, 3D structures were modeled for WT-F and MF-F. Based on structural quality assessment (Table S2), WT_model_5 and MF_model_3 were selected as the optimal models. The MF-F model exhibited favorable stereochemical parameters (Z-score: −10.44) with the majority of residues in favored Ramachandran regions and no significant structural irregularities (Figure S3), supporting its suitability for subsequent docking analyses.

**Figure 2:**
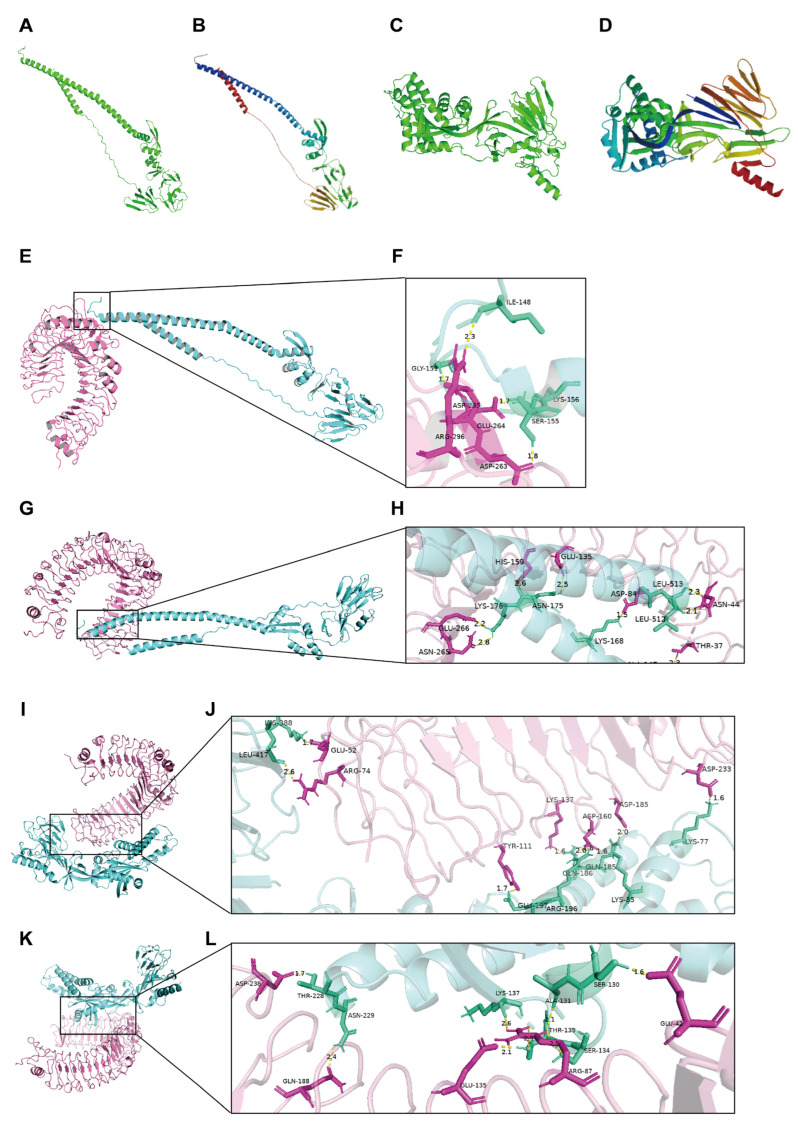
Structure modeling of antigens and molecular docking with TLR2/4. (**A**–**D**) Structure models generated for WT and MF protein with Swiss-Prot toolkit. (**A**,**C**) The templates of F protein monomer were acquired from experimental structures in (**A**) post-F (3RRR) and (**C**) PreF conformation (7KQD), and (**B**,**D**) the models were generated based on corresponding template for both (**B**) WT and (D) MF protein. (**G**–**L**) Models of molecular docking and magnified interface. 4 complexes were generated as (**E**,**F**) WT-TLR2, (**G**,**H**) WT-TLR4, (**I**,**J**) MF-TLR2 and (**K**,**L**) MF-TLR4. The integrative view of models were listed at left, while interfaces magnified at right. Inter-molecule hydrogen bonds were indicated as yellow dash lines and marked with distance. Amino acid residues involving in hydrogen bonds were visualized as green at antigen side and pink at receptor side.

To assess the potential to initiate innate immunity, molecular docking was performed between candidate vaccines and key pattern recognition receptors. Four stable complexes were then generated, namely, WT-TLR2 (Figure 2E), WT-TLR4 (Figure 2G), MF-TLR2 (Figure 2I) and MF-TLR4 (Figure 2K), with the interface magnified alongside (Figure 2F,H,J,L). As the driving forces of interaction, a panel of intermolecular hydrogen bonds and salt bridges formed (Table 2 and Table S3). The stability of the complexes was also supported by energy parameters (Table S4), mainly total energy and binding energy. Changes in protein conformation were mild on the basis of the acceptable value of Δinternal energy for all the complexes (Table S4).

After comprehensive evaluation, distinctiveness emerged in the MF-TLR2 model compared with the other three models, suggesting that the performance was distinguishable after immunization. In silico docking predicted that the MF-TLR2 complex exhibited more extensive interfacial contacts and a greater number of stabilizing interactions compared to WT-TLR2 and other models (Table 2, Table S3 and Table S4), suggesting potentially stronger binding affinity. However, these computational predictions of interface properties require experimental validation through direct TLR activation assays.

### 3.3. MOF-mRNA Molecules Optimized by NLP Algorithms Exhibited Predicted Increased Structural Stability and a Reduced Energy State

**Figure 3:**
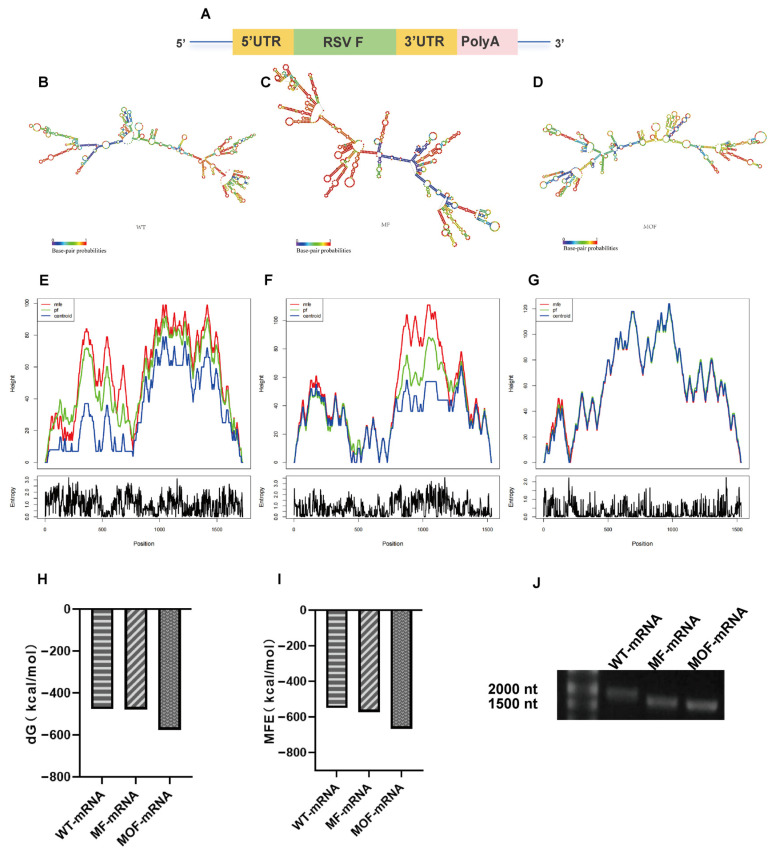
Structural and energetic properties of mRNA molecules. (**A**) Structural schematic of mRNA vaccines, containing 5′ UTR, antigen coding sequence, 3′ UTR, and a poly(A) tail. (**B**–**D**) Secondary structures of mRNA molecules predicted with RNAfold for (**B**) WT, (**C**) MF and (**D**) MOF. (**E**–**G**) Mountain plots generated with RNAfold for predicted structures of (**E**) WT, (**F**) MF and (**G**) MOF to evaluate the properties of stability. Curves of MFE were labeled in red color, thermodynamic ensemble in green and centroid structure in blue, while entropies in black and located below mountain plots. (**H**) Gibbs free energy and (**I**) minimum free energy calculated integratively for each structure of mRNA. (**J**) Denaturing electrophoresis of prepared mRNA molecules on agarose gel.

Based on the unique characteristics of mRNA vaccines, the mRNA encoding the F protein was further designed and modified. Its theoretical secondary structures and energy characteristics were evaluated to provide guidance for subsequent research. On the basis of the designed variant of the RSV antigen, mRNA vaccine candidates were then generated by centering them on the antigen coding sequence and flanking them with a 5′ UTR, 3′ UTR and poly(A) tail (Figure 3A). Primarily, the coding sequences were reverse-translated from the polypeptide sequences of WT-F and MF-F. Hereafter, optimization of the codon usage and secondary structure was carried out for MF mRNA with linear design, a natural language processing algorithm. The MOF mRNA sequence was subsequently generated. Following sequence optimization, 122 bases were adjusted in the MOF relative to the MF, resulting in approximately 92% similarity between the two mRNA sequences (Supplementary Material S2).

Distinct secondary structures were demonstrated through RNAfold simulation (Figure 3B–D). Obviously, broader helical regions, fewer hairpin loops, and fewer internal loop structures were observed in the MOF molecule, indicating greater conformational stability (Figure 3D). With further depiction of the mountain plots for the MFE (minimum free energy) structures (red line), thermodynamic ensembles (green line) and centroid structure (blue line), the curves closely overlapped with each other in the MOF (Figure 3G) but were significantly dispersed in the MF and WT mRNAs (Figure 3E,F), which suggested a unified structure for the MOF molecules and diverged for the rest in an intuitive manner. Corresponding to the extended helical segments found in the predicted secondary structure, longer slopes were observed in the MFE plot of the MOF, while higher peaks suggested tighter base-pairing compactness (Figure 3G, red line). Moreover, higher and more integrated peaks of the thermodynamic ensemble (Figure 3G, green line) reflected a lower diversity of accessible states and higher stability. The calculations revealed the lowest values for the MOF in terms of either dG (Figure 3H) or MFE (Gibbs free energy) (Figure 3I), which suggested its excellent stability. Furthermore, consistent with the predicted tightness of intramolecular compaction, the mobility of the synthesized MOF mRNA was highest in the agarose gel electrophoresis results (Figure 3J).

Taken together, these in silico and in vitro findings suggest that the MOF molecule has favorable structural properties that may enhance intracellular translation of the core antigen.

### 3.4. Enhanced Translation and Presentation of the Prefusion Epitope Were Brought out by the MOF-mRNA Sequence

After the mRNAs were prepared and purified in vitro, quality control was carried out for the mRNA bulk drug substances and final mRNA-LNP products (Figure S4). Comprehensive characterization of the LNPs synthesized in this study demonstrated an encapsulation efficiency > 97% (Figure S4F), a hydrodynamic diameter of 80–90 nm (Figure S4A), a polydispersity index < 0.1 (Figure S4C), and a Zeta potential ranging from −4 to −1 mV (Figure S4D). Additionally, no residues of various key enzymes were detected (Figure S4E). A translucent, light blue emulsion containing a well-encapsulated mRNA-LNPs product was then harvested for each candidate vaccine (Figure S4G). All quality attributes were confirmed to satisfy the predetermined acceptance criteria, thereby validating the suitability of the mRNA-LNPs formulations for downstream vaccine assessment.

**Figure 4:**
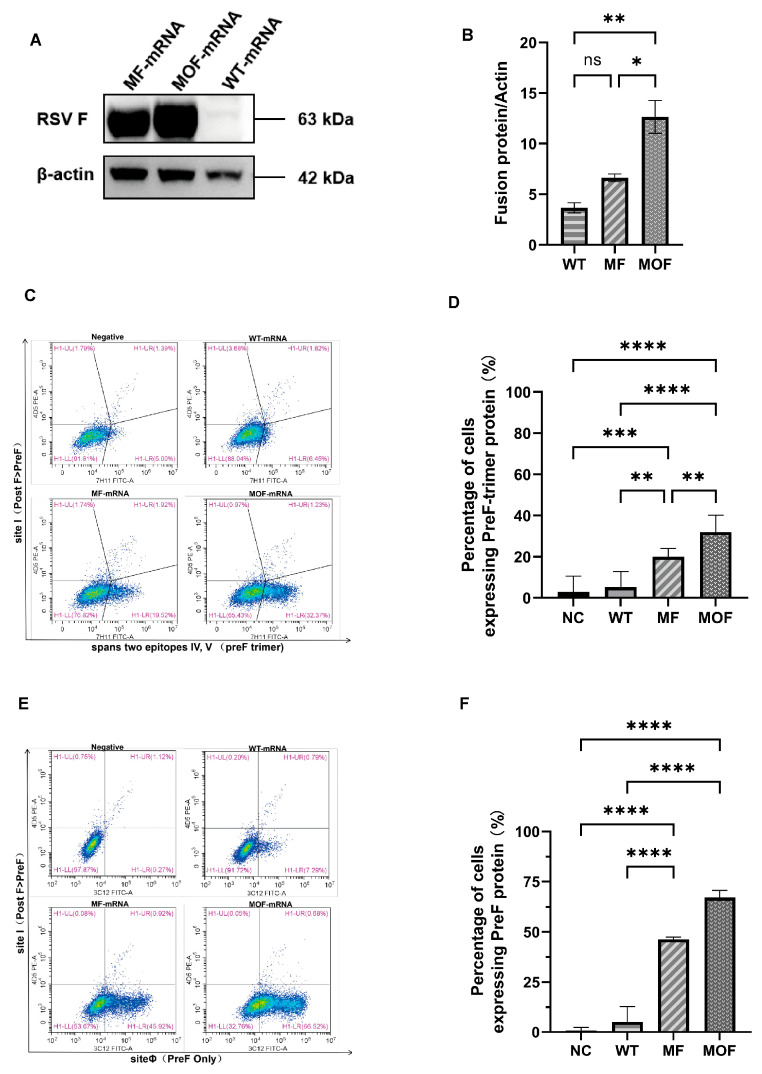
Cells-based expression and presentation of F protein variants. (**A**) Western blot of F proteins in HEK293T cells transfected with indicated mRNAs, with β-actin as internal reference. (**B**) Relative level of F protein quantified based on (**A**) Western blot, normalized with β-actin. (**C**–**F**) Examination of cell-surface presentation of F protein in certain conformation with combined staining of antibody (**C**,**D**) 7H11 targeting site IV, V in PreF trimer or (**E**,**F**) 3C12 targeting site Φ in PreF only with 4D5 to the site I primarily in PostF. Results were presented as the (**C**,**E**) gated flowcytometric plots and (**D**,**F**) corresponding percentages of PreF^+^ cells. N = 3 technical replicates for in vitro studies. Values were showed as means ± SDs. Statistical significance was determined using one-way ANOVA with Tukey’s multiple comparisons test. *, *p* < 0.05; **, *p* < 0.01; ***, *p* < 0.001; ****, *p* < 0.0001; ns: not Significant. The error bars indicate 95% confidence intervals.

The mRNA-LNP products were subsequently transfected into HEK-293 cells. In accordance with the predicted structural advantages, an ~2-fold higher yield of F protein was achieved upon MOF administration than upon the same dose of MF. In contrast, the majority of the unstable F protein from the WT was degraded (Figure 4A,B and Figure S5A).

Owing to the conformational stabilization of the core antigen and structural optimization of its coding mRNA, a high level of PreF trimer was present on the surface of recipient cells of the MF and, furthermore, the MOF RNA vaccine but never on that of the WT control. In brief, cells transfected with the same dose of WT, MF and MOF mRNA were assessed by flow cytometry after being stained with three conformation-specific antibodies. With 7H11 exclusively targeting sites IV and V in the PreF trimer, the positive rate reached 33.60% with MOF, 21.44% with MF and 8.27% with WT (Figure 4C,D). A similar trend was demonstrated for the 3C12 unique recognition site Φ of the PreF conformation, with 67.20%, 46.84% and 8.08% positivity for MOF, MF and WT, respectively (Figure 4E,F). However, the staining level of the 4D5 antibody remained within the range of 1~2% for any mRNA (Figure 4C,E), which indicated the low exposure level of site I and, presumably, the low level of postF conformation.

### 3.5. Robust RSV-Specific Humoral Immunity Was Elicited with the MOF-mRNA Vaccine

**Figure 5:**
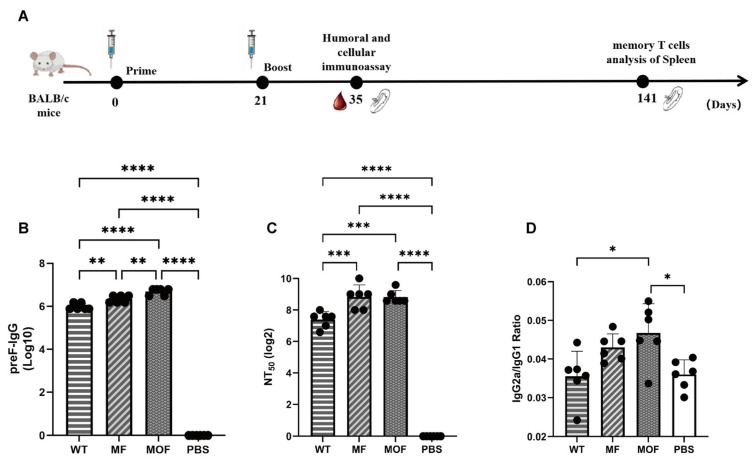
Schematic of vaccination and RSV-specific humoral immunity. (**A**) Scheme of immunization. BALB/c mice at 6–8 wks age were primed and boosted at day 0 and day 21, respectively, at 10 μg/dose/mouse. (**B**–**D**) humoral immunity was analyzed with serum harvested at day 35 (equivalent to 14 days post 2nd immunization) for the levels of (**B**) PreF-specific IgG, (**C**) neutralizing capacity to RSV and (**D**) the ratio of IgG1/IgG2a. N = 6 biologically independent animals per group for in vivo studies. Values were showed as means±SDs. Statistical significance was determined using one-way ANOVA with Tukey’s multiple comparisons test. *, *p* < 0.05; **, *p* < 0.01; ***, *p* < 0.001; ****, *p* < 0.0001. The error bars indicate 95% confidence intervals.

A two-dose schematic was proposed for immunization in BALB/c mice (Figure 5A), with an interval of three weeks and lasting for more than 140 days to the end. For each candidate vaccine, 10 μg per dose was used for each mouse. Two weeks after the boost, serum samples were collected and analyzed.

Compared with those in the PBS control group, robust RSV-specific IgG was elicited by any candidate (Figure 5B). Alternatively, compared with those of the WT candidate, the titers of the MOF and MF candidates were 5- and 2-fold greater, respectively (Figure 5B). Correspondingly, the average level of neutralizing antibody was enhanced approximately 2.6-fold when MOF rather than WT mRNA was used (Figure 5C). In addition, compared with that in the WT group, the IgG2a/IgG1 ratio in the MOF group significantly increased (Figure 5D), which suggested a robust Th1-biased immune response.

Collectively, the results of this study demonstrated that vaccination with MOF mRNA induced stronger RSV-specific humoral immunity compared with MF or WT mRNA in this experimental setting.

### 3.6. Enhanced Th1-Biased T-Cell Responses Were Induced by the MOF-mRNA Vaccine

**Figure 6:**
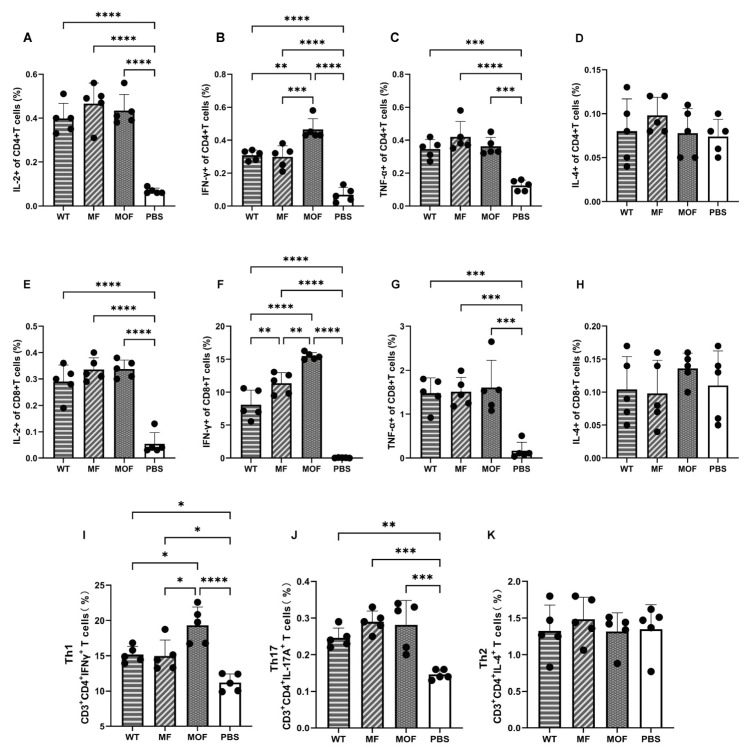
RSV-specific cellular immunity in spleen at 14 days post 2nd immunization. (**A**–**H**) Percentages of cytokine-secreting subsets quantified in (**A**–**D**) CD4^+^ and (**E**–**H**) CD8^+^ T cells respectively. 4 cytokines were checked, including (**A**,**E**) IL-2^+^, (**B**,**F**) IFN-γ^+^, (**C**,**G**) TNF-α^+^ and (**D**,**H**) IL-4^+^ population. (**I**–**K**) Percentages of Th1, (**I**) Th17 (**J**) and (**K**) Th2 cells. N = 5 biologically independent animals per group for in vivo studies. Values were showed as means ± SDs. Statistical significance was determined using one-way ANOVA with Tukey’s multiple comparisons test. *, *p* < 0.05; **, *p* < 0.01; ***, *p* < 0.001; ****, *p* < 0.0001. The error bars indicate 95% confidence intervals.

T-cell immune responses were further evaluated with mouse spleens collected on the same day as serum collection. RSV-specific subsets of T lymphocytes were determined after stimulation with viral peptides. The frequencies of T lymphocytes that secreted IL-2, IL-4, TNF-α, and IFN-γ were then monitored among both helper and cytotoxic T cells (Figure 6A–H). First, compared with those in the PBS control group, the numbers of CD4^+^ and CD8^+^ T cells in the subsets of IL-2-, TNF-α- or IFN-γ-secreting cells markedly increased after two shots of any vaccine candidate (Figure 6A–C,E–G). However, only IFN-γ^+^ CD4^+^ and IFN-γ^+^ CD8^+^ subsets, but not the population that secretes IL-2 or TNF-α, were significantly elevated in the MOF group compared with the MF- or WT-vaccinated group (Figure 6B,F). Specifically, an approximately 20% increase in the number of CD4^+^ T lymphocytes was observed in response to treatment with MOF compared with that in response to treatment with MF or WT (Figure 6B). Similarly, the percentage of CD8^+^ T cells was 15.47% in response to the MOF, 11.34% in response to the MF, and only 8.11% in response to the WT (Figure 6F). On the basis of these data, the use of MOF, an mRNA with an optimized structure and encoding a PreF variant, should have greater potential to promote Th1-biased immunity. In addition, no induction of IL-4 producing T lymphocytes was detected following vaccination with the candidate vaccines, suggesting that Th2-biased immune responses were not predominant in this study. While these data indicate a potentially favorable immunological profile regarding VERD risk, direct assessment of VERD would require challenge studies with histopathological analysis. (Figure 6D,H).

Further confirmation of the generation of Th1, Th2 and Th17 lymphocytes was carried out. Similar to the trends observed in the CD4^+^ or CD3^+^ populations, IFN-γ^+^ CD3^+^ CD4^+^ T cells (Th1) were induced significantly by all three candidates and showed the most pronounced response in the MOF-elicited group rather than in the WT or MF groups (Figure 6I). Furthermore, a significantly increased proportion of IL-17A^+^ (Th17) cells was observed in all vaccinated groups (Figure 6J), which may have contributed to the increase in mucosal defense and neutrophil recruitment, suggesting a potentially favorable immune profile for responding to RSV infection. Importantly, no increase in the number of IL-4^+^ CD3^+^ CD4^+^ T cells (Th2) was detected after the vaccination of any candidate (Figure 6K), which may be consistent with a reduced concern for Th2-dominant VERD risk, though direct challenge studies are required to confirm this.

### 3.7. Proliferation and Differentiation of Memory T Cells Were Effectively Promoted in MOF-mRNA-Vaccinated Mice

**Figure 7:**
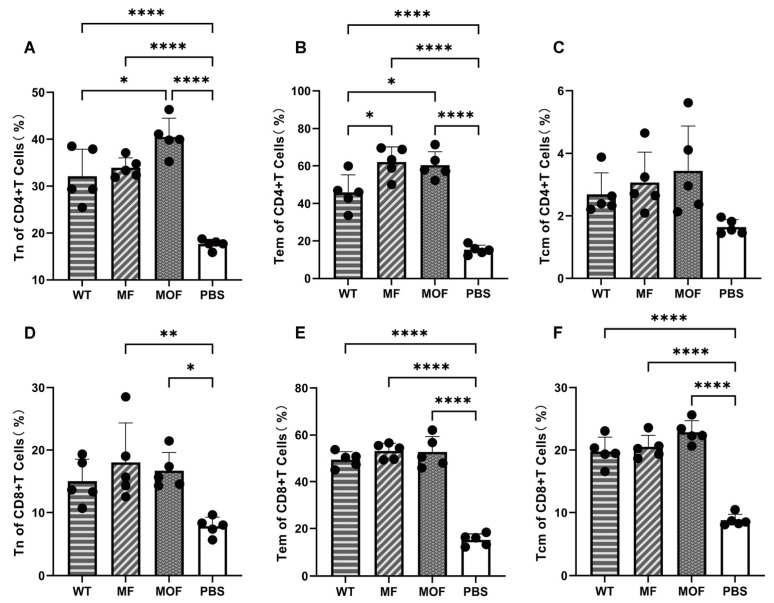
Memory T cell populations evaluated at 120 days post vaccination. (**A**–**F**) Percentages of memory subsets in (**A**–**C**) CD4^+^ and (**D**–**F**) CD8^+^ T cells, including (**A**,**D**) Tn cells, (**B**,**E**) Tem cells and (**C**,**F**) Tcm cells. N = 5 biologically independent animals per group for in vivo studies. Values were showed as means ± SDs. Statistical significance was determined using one-way ANOVA with Tukey’s multiple comparisons test. *, *p* < 0.05; **, *p* < 0.01; ****, *p* < 0.0001. The error bars indicate 95% confidence intervals.

To determine the percentage of various memory T lymphocyte subtypes, including naïve T cells, central memory T cells, and effector memory T cells, spleens were harvested from mice immunized with three candidate vaccines at 120 days after the boost. As determined by flow cytometry, the percentages of naïve T cells among CD4^+^ T cells were 32.09% for the WT, 33.88% for the MF, and 40.48% for the MOF, whereas they were 17.59% in the PBS control; these values increased by approximately 1.8-, 1.9- and 2.3-fold, respectively (Figure 7A). Enhancement of effector memory T cells was also observed in CD4^+^ T cells when the percentages of cells in the MOF and MF groups were compared with those in the WT group, which were 62.09% and 60.32% versus 45.83%, respectively (Figure 7B). In CD8^+^ T cells, similar trends were detected for naïve and effector memory cells (Figure 7D,E). For central memory T cells, significant disparities were noted in CD8^+^ but not CD4^+^ T cells. Compared with those of the PBS control, 10.98%, 11.72%, and 14.08% increases were achieved by WT, MF and MOF, respectively (Figure 7F).

Collectively, all three mRNA vaccine candidates effectively induced the proliferation and differentiation of memory T lymphocytes, among which MOF showed the best robustness. These results provide important information for the development and maintenance of long-term memory T-cell responses following RSV vaccination.

## 4. Discussion

In the current study, an mRNA vaccine was designed to encode a conformationally stabilized variant of the RSV F protein. This candidate induced RSV-specific humoral and cellular immunity. Notably, it elicited an enhanced Th1 bias and memory T-cell subpopulations.

To robustly induce neutralizing antibodies, a DS-Cav1 variant was used as a reference in the design of the core antigen MF. As demonstrated previously, neutralizing epitopes were highly exposed in the DS-Cav1 variant, and immune protection was then robust [46]. To generate MF mutants, multiple mutations [47] (e.g., A149C and Y458C) were introduced into the DS-Cav1 scaffold to link the monomers with additional disulfide bonds. Despite the performance of DS-Cav1 and its related variants [46], the MF variant was maintained as a trimer in the PreF conformation, as demonstrated by structural modeling and flow cytometric analysis. In addition, in silico analysis revealed that the physicochemical and immunological properties of the MF mutant and wild-type F protein were comparable. Moreover, the potential for robust binding between the MF protein and pattern recognition receptors such as TLR2 or TLR4 was predicted from the molecular docking and dynamic simulation results. These interactions play important roles in the initiation of the host immune response. In summary, the properties of the MF variant, rather than the wild-type F protein, provide a prominent basis for the development of an RSV vaccine.

Accumulating evidence has established mRNA vaccines as a new option for vaccine formulation and delivery. To encode the core antigen MF, the MOF mRNA sequence was then generated after comprehensive optimization with the NLP algorithm, from which better molecular stability and higher translational efficiency were subsequently expected. NLP-based mRNA optimization overcomes the limitations of conventional codon optimization by employing a multi-objective framework, in which the design is converted into an optimal path search in a deterministic finite automaton (DFA). Structural stability and expression efficiency are simultaneously optimized, markedly improving mRNA half-life, protein expression, and immunogenicity. The core mechanism involves increased double-stranded content and reduced degradation sites, achieving a balance between stability and translational efficiency [38]. In silico predictions suggested that the MOF mRNA has a lower energy profile, greater compactness, and stronger thermostability than non-optimized mRNAs of MF or WT. These predictions were supported by the higher electrophoretic mobility observed on agarose gels. Coupled with codon usage being biased toward human cells, a reinforced expression capacity was therefore achieved with the use of MOF, which was validated by Western blotting.

Combining the rational design of the core antigen and AI facilitated the optimization of coding sequences, and the functional characteristics of the candidate vaccine were then rooted and subsequently demonstrated in animal experiments.

In the assessment of the humoral immune response, compared with the WT candidate, the candidate vaccines MF and MOF elicited stronger antibody reactions to the PreF conformation and neutralizing effects on RSV. These results were consistent with the robust stabilization of the PreF conformation and the exposure of neutralizing epitopes in DS-Cav1-based variants [48] and agreed with the flow cytometric evaluation of the core antigen. Thus, stabilizing the PreF conformation is considered a critical factor in RSV vaccine development [49]. Furthermore, in our study, the PreF-expressing mRNA vaccine elicited RSV-binding antibody titers of approximately 10^6^ and neutralizing antibody titers of >2^8^. While these values are within a similar range to those reported for a PreF subunit vaccine by Chen et al. (approximately 10^5^ and 2^7.06^, respectively) [50], direct cross-study comparisons are limited by differences in experimental designs and require further validation through direct experimental comparison. Additionally, within our study, significantly higher titers of RSV-specific binding antibodies and stronger Th1-biased T-cell responses were detected in mice treated with the MOF vaccine candidate compared with those treated with the MF and WT candidates. This finding indicates that structural optimization of the mRNA sequence contributes to improved immunogenicity.

For the cellular immunity, however, limited evidence is available from studies of RSV PreF subunit vaccines. In principle, the intracellular translational nature of mRNA vaccines enhances CTL immune responses, which are mediated by the MHC I antigen presentation pathway. In this study, compared with the MF and WT, the MOF candidate vaccine induced increased Th1 and Th17 responses. However, the three candidate vaccines did not significantly affect the Th2 response. This trend contrasts with the Th2-skewed immune response induced by earlier inactivated RSV vaccine, which is closely associated with vaccine-enhanced respiratory disease (VERD) [51]. This may also be attributed to, at least partially, the avoidance of unexpected conformational disruptions unavoidable during in vitro purification, which may reduce immunogenicity and induce VERD-associated adverse reactions. Notably, the proportions of IFN-γ^+^ CD4^+^/IFN-γ^+^ CD8^+^ T cells targeting RSV were significantly higher in the MOF group than in either the WT or MF groups, suggesting that this vaccine candidate more effectively activates and expands these cells.

Despite the European Commission approval granted to Moderna for its RSV vaccine mRESVIA^®^ [52], the maintenance of long-term immune protection has been recognized as a key limitation among currently available RSV vaccines. Encouragingly, the MOF vaccine candidate exhibited promising immunological characteristics that may address this limitation. However, head-to-head comparative studies with approved vaccines are required to confirm any potential advantages. After the mice were vaccinated with MOF, elevated levels of naïve CD4^+^ T cells (Tn) and effector memory CD4^+^ T cells (Tem) were detected in the spleen. On the other hand, central memory CD4^+^ T cells (Tcm) were present at similar levels across groups, which may partly explain the notable decrease in vaccine-induced antibody levels over time.

The generation of these memory T cell populations is a promising indicator of long-term immunological memory, a key goal of next-generation RSV vaccines [53].

Additionally, it must be acknowledged that direct viral challenge experiments in gold-standard animal models, such as cotton rats, are required in future studies to further validate vaccine efficacy and safety. Histopathological analysis of lung tissues post-challenge in these models is particularly necessary for definitive assessment of VERD risk. Meanwhile, the explosion of AI has brought new prospects to biomedicine, especially the field of vaccine design. However, it may also be accompanied by certain safety issues that require scientific application [54].

## 5. Conclusions

In summary, our vaccine design exhibited several favorable immunological features. First, rational design of the core antigen for conformational stabilization was applied. Second, the mRNA vaccine platform was utilized. Finally, an AI tool was employed to facilitate the structural optimization of the mRNA vaccine. With this strategy, substantial production of RSV-specific neutralizing antibodies, enhanced RSV-specific Th1-biased cellular immunity, and the potential to sustain immune memory were observed. Overall, a balance of effectiveness and safety may be achievable with the MOF vaccine candidates generated in the current study. However, the potential for improved control of side effects, particularly VERD, remains speculative and awaits confirmation in challenge and pathology experiments.

## Figures and Tables

**Table 1:** Properties of the two proteins in physicochemistry and immunology.

Index	WT-F	MF-F
No.of amino acids	574	510
Molecular weight	63 kDa	56 kDa
Theoretical pI	9.17	8.28
Instability index	39.78 (Stable)	38.53 (Stable)
Estimated half-life	30 h (mammalian reticulocytes, in vitro) >20 h (yeast, in vivo) >10 h (*E. coli*, in vivo)	
Aliphatic index	101.15	102.18
GRAVY	−0.01	0.075
Allergenicity (AllerTOP)	No	No
Antigenicity (VaxiJen)	0.5011	0.4838
Antigenicity (ANTIGENPro)	0.9046	0.8834
Solubility (SolPro)	0.6944	0.7466
Solubility (Protein-Sol)	0.490	0.430

**Table 2:** Interface of interactions between the WT/MF and TLR2/TLR4.

Docking Model	Chain	Interface			Salt Bridges	Hydrogen Bonds
		Residius	Atoms	Area (Å^2^)		
WT-TLR2	Vaccine	13	48	566.2	2	6
	Receptor	20	66	468.3		
WT-TLR4	Vaccine	22	73	968.6	3	6
	Receptor	35	104	921.2		
MF-TLR2	Vaccine	39	117	1105.1	6	8
	Receptor	35	116	1182.6		
MF-TLR4	Vaccine	40	113	997.0	1	2
	Receptor	30	121	1019.6		

## Data Availability

All data supporting the findings of this study are available within the manuscript. Any additional data are available from the corresponding author upon reasonable request.

## Supplementary Materials

The following supporting information can be downloaded at: https://www.mdpi.com/article/10.3390/vaccines14040356/s1, Supplementary Materials: S1 (supplementary experimental results and raw data), S2 (information on relevant mRNA and protein sequences), and S3 (exact P-values in the statistical analysis results).

## Appendix A

In this study, we successfully designed and produced an mRNA vaccine encoding the RSV prefusion F (PreF) protein. The properties of the PreF antigen have been widely evaluated in silico, and the mRNA coding sequences have been optimized using NLP algorithms, which suggests improved mRNA structural stability and translational efficiency based on in vitro and in vivo assays. The antigen expressed in mammalian cells exhibited a correct conformation and retained key antigenic epitopes. In a murine model, the vaccination regimen induced a balanced and potent Th1-biased immune response, generating high levels of neutralizing antibodies and antigen-specific T-cell immunity. It also promoted the proliferation and differentiation of memory T cells effectively, indicating its potential to confer long-term immunological memory. These findings represent a promising step forward in the pursuit of an effective RSV vaccine and suggest the potential of the developed mRNA vaccine to induce substantial immune responses that may correlate with protection in future challenge studies.
