# Supplementary material for: Enhanced Th1 Cellular Immunity Induced by an RSV-F mRNA Vaccine Rationally Designed Using NLP Algorithms
Source: Vaccines (Basel). 2026 Apr 16;14(4):356. doi: 10.3390/vaccines14040356 (PMC13119773; doi:10.3390/vaccines14040356)
Supplement: Supplementary file 1 [file vaccines-14-00356-s001.zip › Supplementary Material S1.pdf]

Supplementary Material S1

```
          1        10        20        30        40        50        60
WT        MELLILKANAITTILTAVTFCFASGQNITEEFYQSTCSAVSKGYLSALRTGWYTSVITIE
MF        MELLILKANAITTILTAVTFCFASGQNITEEFYQSTCSAVSKGYLSALRTGWYTSVITIE

                   70        80        90       100       110       120
WT        LSNIKKNKCNGTDAKIKLIKQELDKYKNAVTELQLLMQSTPATNNRARRELPRFMNYTLN
MF        LSNIKENKCNGTDAKVKLIKQELDKYKNAVTELQLLMQSTPAT.................

                  130       140       150       160       170       180
WT        NAKKTNVTLSKKRKRRFLGFLLGVGSAIASGVAVSKVLHLEGEVNKIKSALLSTNKAVVS
MF        .......................GSGSAICSGVAVCKVLHLEGEVNKIKSALLSTNKAVVS

                  190       200       210       220       230       240
WT        LSNGVSVLTSKVLDLKNYIDKQLLPIVNKQSCSISNIETVIEFQQKNNRLLEITREFSVN
MF        LSNGVSVLTFKVLDLKNYIDKQLLPILNKQSCSISNIETVIEFQQKNNRLLEITREFSVN

                  250       260       270       280       290       300
WT        AGVTTPVSTYMLTNSELLSLINDMPITNDQKKLMSNNVQIVRQQSYSIMSIIKEEVLAYV
MF        AGVTTPVSTYMLTNSELLSLINDMPITNDQKKLMSNNVQIVRQQSYSIMCIIKEEVLAYV

                  310       320       330       340       350       360
WT        VQLPLYGVIDTPCWKLHTSPLCTTNTKEGSNICLTRTDRGWYCDNAGSVSFFPQAETCKV
MF        VQLPLYGVIDTPCWKLHTSPLCTTNTKEGSNICLTRTDRGWYCDNAGSVSFFPQAETCKV

                  370       380       390       400       410       420
WT        QSNRVFCDTMNSLTLPSEVNLCNVDIFNPKYDCKIMTSKTDVSSSVITSLGAIVSCYGKT
MF        QSNRVFCDTMNSRTLPSEVNLCNVDIFNPKYDCKIMTSKTDVSSSVITSLGAIVSCYGKT

                  430       440       450       460       470       480
WT        KCTASNKNRGIIKTFSNGCDYVSNKGVDTVSVGNTLYYVNKQEGKSLYVKGEPIINFYDP
MF        KCTASNKNRGIIKTFSNGCDYVSNKGVDTVSVGNTLYCVNKQEGKSLYVKGEPIINFYDP

                  490       500       510       520       530       540
WT        LVFPSDEFDASISQVNEKINQSLAFIRKSDELLHNVNAVKSTTNIMITTIIIVIIVILLS
MF        LVFPSDEFDASISQVNEKINQSLAFIRKSDELLHNVNAGKSTTNIMITTIIIVIIVILLS

                  550       560       570
WT        LIAVGLLLYCKARSTPVTLSKDQLSGINNIAFSN
MF        LIAVGLLLY.........................
```

Figure S1. Alignment of WT and MF amino acid sequences.

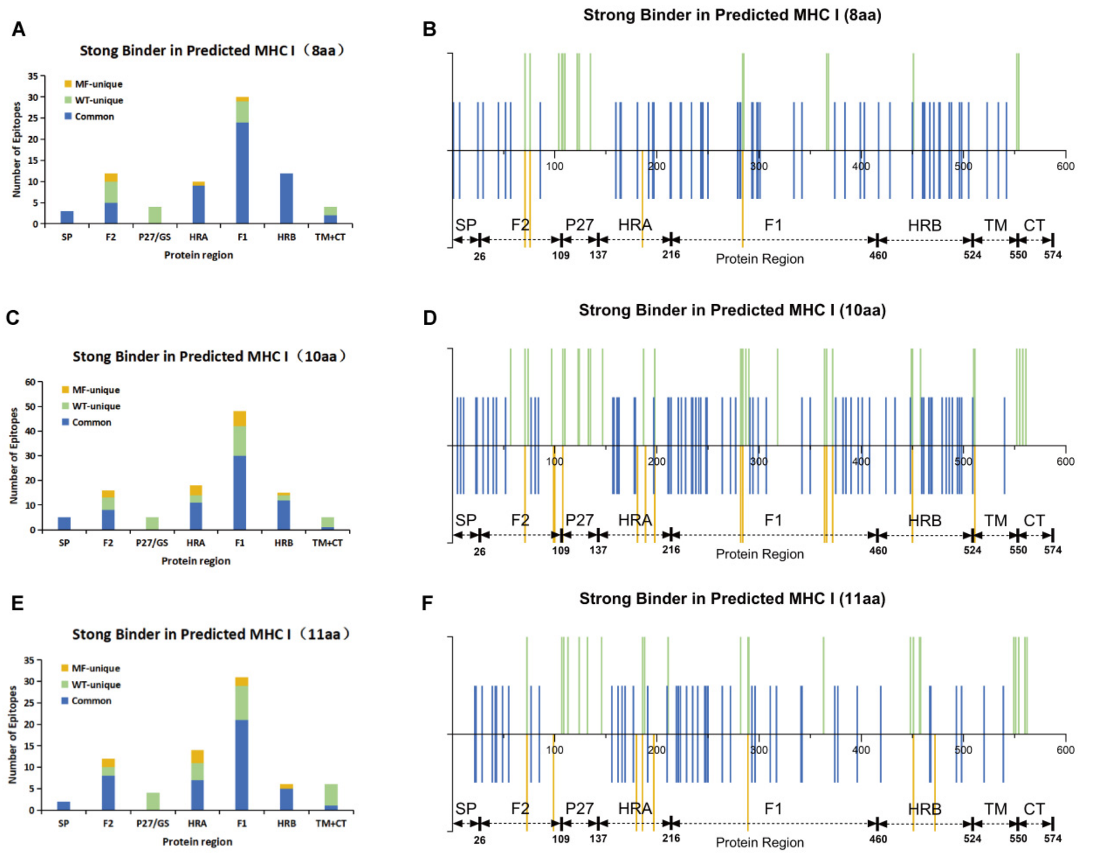

Figure S2. Distribution of T cell class I epitopes across structural domains and comparing between WT and MF proteins. Predicted T cell class I epitopes showed strong binding to MHC molecules at length of (A, B) 8aa, (C, D) 10aa, and (E, F)11aa. In left column (A, C and E), number of epitopes was summarized in each domain; while in right column (B, D and F), distribution of epitopes was aligned between WT and MF proteins. The epitopes common for both proteins were indicated in blue, epitopes unique to WT protein in light green, and epitopes unique to MF protein in orange-yellow.

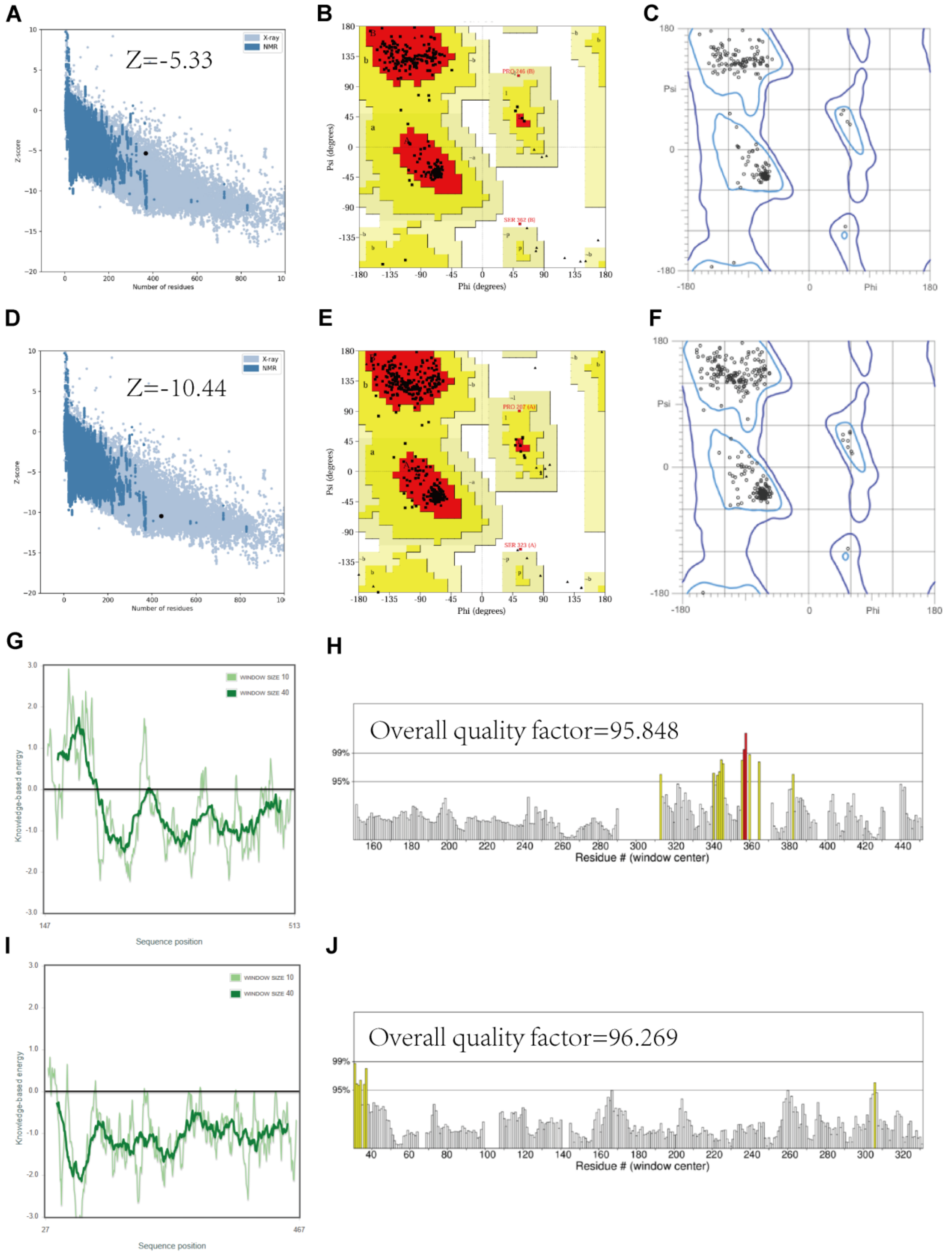

Figure S3. Quality assessment of structural models for WT and MF proteins . (A-F) The overall quality evaluation for structural models of (A-C) WT protein and (D-F) MF protein, with (A, D) ProSA-web tool and (B, C and E, F) Ramachandran plots depicted from (B, E) PROCHECK or (C, F) MolProbity. Z-scores generated with ProSA-web were labeled in (A, D) plots. (G-J) The local evaluation of model quality for (G, H) WT protein and (I, J) MF protein. (G, I) Local energy level was calculated with ProSA-web tool for fragments one-by-one. (H, J) Probability of local irrationality was quantified with ERRAT2 tool based on the (B, E) Ramachandran plots, with overall quality factor scored and labeled.

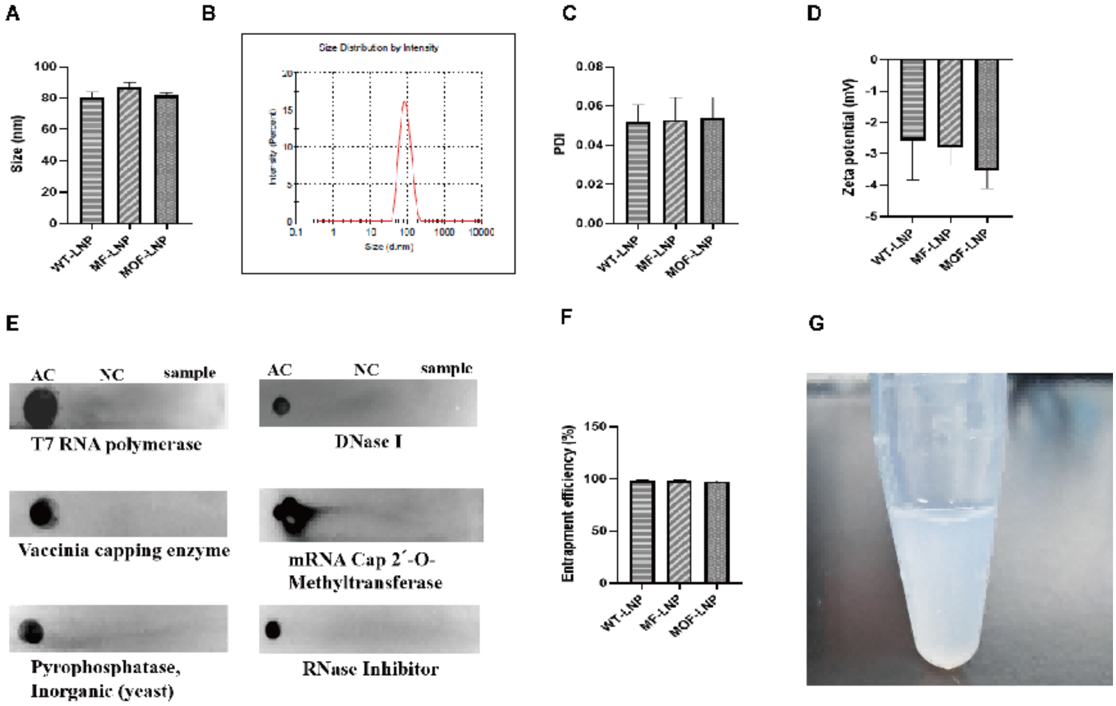

Figure S4. Quality control in mRNA vaccine manufacturing. (A-D) the evaluation of mRNA quality for three candidate vaccines with Malvern particle size analyzer, including (A) The particle size and aggregation degree with (B) one representative peak plot of the mRNA, (C) PDI and (D) zeta potential. (E) Residual content of enzyme used in preparation of mRNA bulk drug substance quantified with dot blot. (F) The encapsulation efficiency of the mRNA-LNPs assessed using RNA Qubit method. (H) The final mRNA-LNP vaccine product, a translucent light blue emulsion.

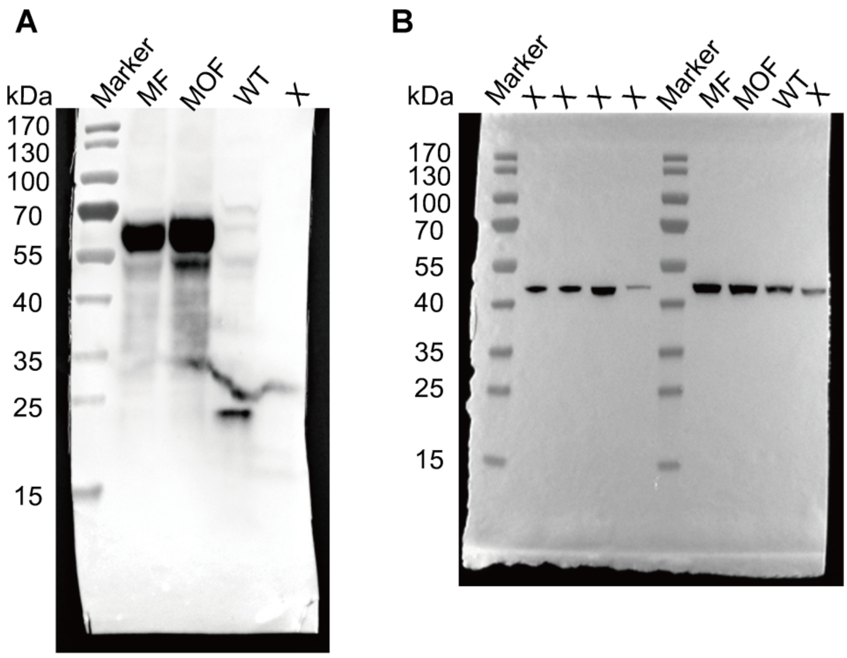

Figure S5. Original images of Western blot. (A) F protein expression and (B) β-actin as internal reference.

Table S1 Summary of T epitope prediction results

			WT	MF	Common	WT-unique	MF-unique
	SUM	Strong Binder	447	372	-	-	-
		MHC	10395	9975	-	-	-
		Epitope-MHC	427881	403890	-	-	-
MHC-I	8aa	Strong Binder	72	60	56	16	4
		MHC	9958	9330	-	-	-
		Epitope-MHC	61295	47671	-	-	-
	9aa	Strong Binder	210	179	151	59	28
		MHC	8276	9975	-	-	-
		Epitope-MHC	220631	247333	-	-	-
	10aa	Strong Binder	98	81	67	31	14
		MHC	10228	9730	-	-	-
		Epitope-MHC	87463	67857	-	-	-
	11aa	Strong Binder	67	52	44	23	8
		MHC	9669	8889	-	-	-
		Epitope-MHC	58492	41029	-	-	-
MHC-II		Strong Binder	130	96	76	54	20
		MHC	6774	6774	-	-	-
		Epitope-MHC	77948	53567	-	-	-

Note: The table provides a detailed enumeration of the number of epitopes, with SB indicating epitopes exhibiting strong binding.

Table S2 Scoring of the Swiss-prot model

Antigen	Model ID	GDT-HA	RMSD	MolProbity	Clash score	Poor rotamers	Rama favored
WT	**Initial**	1.0000	0.000	1.591	2.5	2.1	95.3
	**model_1**	0.9612	0.380	1.349	6.3	0.3	98.9
	**model_2**	0.9571	0.401	1.296	5.5	0.3	98.1
	**model_3**	0.9537	0.416	1.307	5.7	0.6	99.2
	**model_4**	0.9632	0.381	1.317	5.8	0.0	98.6
	**model_5**	0.9728	0.367	1.273	5.1	0.6	98.4
MF	**Initial**	1.0000	0.000	1.871	4.1	2.7	95.0
	**model_1**	0.9915	0.284	1.610	12.6	0.2	98.9
	**model_2**	0.9870	0.297	1.553	10.9	0.5	98.9
	**model_3**	0.9853	0.316	1.548	10.7	0.7	99.1
	**model_4**	0.9904	0.287	1.578	11.6	0.2	98.6
	**model_5**	0.9841	0.299	1.653	14.0	0.2	99.1

Note: GDT-HA (averaging>0.95) for overall accuracy, RMSD (averaging <0.42 Å) for structural deviation, MolProbity Score (excellent level) for stereochemical evaluation, Clash Score for atomic conflict detection, Poor Rotamers (averaging near 0) for side chain conformation assessment, and Rama Favored (averaging>98%) for backbone conformation analysis. All model metrics demonstrated outstanding performance, thus the WT model_5 and MF model_3 with superior comprehensive performance were highlighted in yellow and selected for further in-depth analysis.

Table S3. Hydrogen bonds and salt bridges within vaccine-receptor interface

Docking model	Type of interaction	Residues of interaction (vaccine-receptor)	Distace (Å)
WT | TLR2	Hydrogen bonds	GLY 151-GLN 268	3.86
		GLY 151-GLU 264	2.71
		SER 155-ASN 290	3.38
		LYS 156-ASP 235	2.32
		ILE 148-ARG 296	2.34
		GLY 151-PHE 237	3.83
	Salt bridges	LYS 156-ASP 235	2.56
		LYS 156-ASP 235	2.76
WT | TLR4	Hydrogen bonds	GLU 161-GLN 39	3.20
		LYS 168-ASP 84	1.54
		ASN 175-GLU 135	2.48
		LYS 176-GLU 266	2.18
		LEU 512-ASN 44	2.15
		LEU 513-ASN 44	2.34
	Salt bridges	LYS 168-ASP 84	3.52
		LYS 168-ASP 84	2.58
		LYS 176-GLU 266	3.13
MF | TLR2	Hydrogen bonds	LYS 388-GLU 52	1.74
		LYS 85-ASP 160	1.58
		ARG 196-ASP 160	2.01
		ARG 196-ASP 160	1.64
		GLN 185-ASP 185	2.02
		LYS 77-ASP 233	1.56
		GLN 186-LYS 137	1.64
		GLU 197-TYR 111	1.70
	Salt bridges	LYS 388-GLU 52	3.96
		LYS 388-GLU 52	2.59
		LYS 85-ASP 160	2.60
		ARG 196-ASP 160	2.86
		ARG 196-ASP 160	2.62
		LYS 77-ASP 233	2.58
MF | TLR42	Hydrogen bonds	ASN 229-GLN 188	2.42
		ALA 131-ARG 87	2.14
	Salt bridges	LYS 129-GLU 42	3.63

Table S4 Energy parameters of antigen-receptor interaction in complexes

Energy parameters	Docking Model			
	WT-TLR2	WT-TLR4	MF-TLR2	MF-TLR4
Total energy	-172.326	-179.847	-387.83	-189.109
Binding energy	-131.076	-107.32	-319.07	-134.022
Energy of vdw	-26.1867	-53.6539	-45.3264	-59.8391
Energy of elec	-187.696	-205.993	-373.288	-188.095
Desolvation Energy	-1.91563	-4.34139	11.1327	4.75419
Energy of air	41.5572	79.7994	30.784	58.8252
air violations	2	2	1	1
air rms-dev (Å)	0.468914	1.08924	0.462807	2.72559
Internal energy (free molecules)	-29875	-29942.4	-34238.4	-34586.7
Internal energy (complex)	-29790.3	-29785.7	-34150	-34477.5
Δ Internal energy	84.7	156.7	88.4	109.2

Note: Unit for all energy parameters is kcal/mol.
