# Supplementary material for: Enhanced Th1 Cellular Immunity Induced by an RSV-F mRNA Vaccine Rationally Designed Using NLP Algorithms
Source: Vaccines (Basel). 2026 Apr 16;14(4):356. doi: 10.3390/vaccines14040356 (PMC13119773; doi:10.3390/vaccines14040356)
Supplement: Supplementary file 1 [file vaccines-14-00356-s001.zip › Supplementary Material S2.pdf]

**Supplementary Material S2**

**The open reading frame (ORF) nucleic acid sequence of the WT mRNA**

AUGGAACUGCUGAUCCUGAAGGCCAACGCUAUCACCACCAUCCUGACCG

CCGUGACCUUCUGCUUCGCCAGCGGCCAGAACAUCACAGAAGAAUUCUA

CCAGUCUACCUGUUCUGCAGUGUCUAAGGGCUACCUAAGCGCCCUGAGA

ACCGGAUGGUACACCAGCGUGAUCACAAUCGAGCUGAGCAAUAUCAAG

AAGAACAAGUGUAAUGGUACAGACGCCAAGAUCAAACUGAUUAAGCAA

GAGCUGGACAAGUACAAGAACGCCGUCACAGAGCUGCAGCUGCUGAUG

CAGAGCACCCCUGCCACCAACAACCGGGCCAGACGGGAACUCCCCAGAU

UCAUGAACUACACCCUGAACAACGCCAAGAAGACUAACGUGACCCUGUC

UAAGAAAAGAAAGCGAAGAUUCCUGGGCUUCCUGUUGGGCGUGGGAUC

UGCCAUCGCCUCUGGAGUGGCCGUGUCCAAGGUGCUGCACCUGGAAGGC

GAAGUAAACAAGAUCAAGAGCGCCCUGCUGAGCACAAAUAAAGCCGUC

GUGAGCCUGAGCAACGGCGUCAGCGUGCUGACCAGCAAGGUGUUGGAU

CUGAAAAACUACAUCGAUAAGCAGCUGCUGCCUAUCGUGAACAAGCAG

AGCUGCAGCAUCUCCAACAUUGAGACAGUGAUCGAAUUUCAGCAGAAG

AACAAUCGGCUGCUCGAGAUCACCAGAGAGUUCAGCGUUAAUGCUGGC

GUGACCACCCCAGUGUCCACCUACAUGCUGACCAAUAGCGAGCUGCUCA

GCCUCAUCAACGACAUGCCUAUUACAAAUGAUCAGAAAAAGCUGAUGA

GCAACAACGUGCAGAUCGUGCGGCAGCAAAGCUACAGCAUCAUGAGCA

UCAUCAAGGAAGAGGUGCUGGCCUACGUGGUGCAACUGCCUCUGUACG

GCGUUAUCGACACCCCUUGCUGGAAGCUGCACACCUCUCCUCUGUGUAC

CACCAACACAAAGGAGGGCAGUAACAUCUGCCUGACCCGCACCGAUAGA

GGCUGGUAUUGCGACAACGCCGGCAGCGUGAGCUUCUUCCCUCAGGCUG

AAACCUGCAAGGUGCAGUCCAAUAGAGUGUUCUGCGACACAAUGAACA

GCCUGACCCUGCCUAGCGAGGUCAACCUGUGCAACGUGGACAUCUUCAA

CCCCAAGUACGACUGCAAGAUCAUGACCUCCAAAACAGAUGUGUCCAGC

AGCGUGAUCACAAGCCUGGGCGCCAUCGUGUCCUGCUACGGCAAGACAA

AGUGCACUGCCAGCAACAAGAACAGAGGCAUCAUCAAGACCUUUAGCA

ACGGCUGUGACUACGUGUCCAACAAGGGAGUCGACACCGUGUCGGUGG

GCAACACCCUGUACUACGUGAAUAAACAGGAGGGAAAGAGCCUGUACG

UGAAGGGCGAGCCAAUCAUCAACUUCUACGACCCCCUGGUGUUCCCCAG

CGACGAGUUUGAUGCCUCUAUUAGCCAGGUGAAUGAGAAGAUCAACCA

GAGCCUGGCUUUUAUCCGGAAGUCUGAUGAGCUGCUGCACAACGUCAA

CGCCGUGAAAAGCACAACCAACAUCAUGAUCACCACAAUCAUCAUCGUG

AUCAUCGUGAUCCUGCUGAGCCUGAUCGCCGUGGGCCUGCUGCUUUAU

UGUAAAGCUAGAAGCACUCCUGUGACACUGUCUAAGGACCAGCUGAGC

GGCAUCAACAAUAUCGCCUUCAGCAACUAA

**The corresponding amino acid sequence of the WT mRNA**

MELLILKANAITTILTAVTFCFASGQNITEEFYQSTCSAVSKGYLSALRTGWYT

SVITIELSNIKKNKCNGTDAKIKLIKQELDKYKNAVTELQLLMQSTPATNNRA

RRELPRFMNYTLNNAKKTNVTLSKKRKRRFLGFLLGVGSAIASGVAVSKVLH

LEGEVNKIKSALLSTNKAVVSLSNGVSVLTSKVLDLKNYIDKQLLPIVNKQSC

SISNIETVIEFQQKNNRLLEITREFSVNAGVTTPVSTYMLTNSELLSLINDMPIT

NDQKKLMSNNVQIVRQQSYSIMSIIKEEVLAYVVQLPLYGVIDTPCWKLHTSP

LCTTNTKEGSNICLTRTDRGWYCDNAGSVSFFPQAETCKVQSNRVFCDTMNS

LTLPSEVNLCNVDIFNPKYDCKIMTSKTDVSSSVITSLGAIVSCYGKTKCTASN

KNRGIIKTFSNGCDYVSNKGVDTVSVGNTLYYVNKQEGKSLYVKGEPIINFY

DPLVFPSDEFDASISQVNEKINQSLAFIRKSDELLHNVNAVKSTTNIMITTIIIVII

VILLSLIAVGLLLYCKARSTPVTLSKDQLSGINNIAFSN

**The open reading frame (ORF) nucleic acid sequence of the MF mRNA**

AUGGAGCUGCUGAUCCUGAAGGCCAACGCCAUCACGACCAUCCUGACCG

CCGUGACCUUCUGCUUCGCCAGCGGGCAGAACAUCACCGAGGAGUUCUA

CCAGUCCACCUGCUCCGCCGUGAGCAAGGGCUACCUGUCUGCCCUGAGA

ACCGGCUGGUACACCAGCGUGAUCACCAUCGAGCUGUCCAACAUCAAGG

AGAACAAGUGCAACGGCACCGACGCCAAGGUGAAGCUGAUCAAGCAGG

AGCUGGACAAGUACAAGAACGCAGUGACCGAGCUGCAGCUGCUGAUGC

AGAGCACACCAGCCACCGGUAGCGGGUCCGCCAUUUGCUCCGGCGUGGC

CGUGUGCAAGGUGCUGCACCUGGAGGGCGAGGUGAACAAGAUCAAGAG

CGCCCUGCUCUCCACCAACAAGGCCGUGGUGAGCCUGAGCAACGGGGUG

AGCGUGCUGACCUUCAAGGUGCUGGACCUGAAGAACUACAUCGACAAG

CAGCUGCUGCCUAUCCUGAACAAGCAGAGCUGCAGCAUCAGCAACAUCG

AGACCGUGAUCGAGUUCCAGCAGAAGAACAACCGGCUGCUGGAGAUCA

CCAGGGAGUUCAGCGUGAACGCAGGGGUGACCACACCCGUGUCCACCUA

CAUGCUGACCAACUCCGAGCUGCUGAGCCUGAUCAACGAUAUGCCCAUC

ACCAACGACCAGAAGAAGCUGAUGAGCAACAACGUGCAGAUCGUGCGG

CAGCAGUCCUACUCCAUCAUGUGCAUCAUCAAGGAGGAGGUGCUGGCC

UACGUGGUGCAGCUGCCCCUGUACGGCGUGAUCGACACCCCUUGCUGGA

AGCUGCACACCAGCCCUCUGUGCACCACCAACACGAAGGAGGGCAGCAA

UAUCUGCCUGACCCGGACCGACAGGGGCUGGUACUGCGACAACGCCGGC

AGCGUGUCCUUCUUUCCCCAGGCCGAGACCUGCAAGGUGCAGUCCAACA

GGGUGUUCUGCGACACCAUGAACUCUCGCACCCUGCCCAGCGAGGUGAA

CCUGUGCAACGUGGACAUCUUCAACCCCAAGUACGACUGCAAGAUCAUG

ACCUCCAAGACCGACGUGUCCUCUAGCGUUAUCACCUCCCUGGGCGCCA

UCGUGAGCUGCUACGGCAAGACCAAGUGCACCGCCAGCAACAAGAACAG

GGGCAUCAUCAAGACCUUCAGCAACGGGUGCGACUACGUGUCCAACAA

GGGCGUGGACACCGUGUCCGUGGGCAACACCCUGUACUGCGUGAACAA

GCAGGAGGGCAAGAGCCUGUACGUGAAGGGCGAGCCCAUCAUCAACUU

CUACGACCCUCUGGUGUUCCCCAGCGACGAGUUCGACGCCAGCAUCUCC

CAGGUGAACGAGAAGAUCAACCAGAGCCUGGCCUUCAUCCGCAAGAGC

GACGAGCUGCUGCACAACGUGAACGCCGGCAAGAGCACCACAAACAUCA

UGAUCACCACCAUCAUCAUCGUGAUAAUCGUGAUCCUGCUGUCCCUGAU

CGCUGUGGGCCUGCUGCUGUACUAA

**The corresponding amino acid sequence of the MF mRNA**

MELLILKANAITTILTAVTFCFASGQNITEEFYQSTCSAVSKGYLSALRTGWYT

SVITIELSNIKENKCNGTDAKVKLIKQELDKYKNAVTELQLLMQSTPATGSGS

AICSGVAVCKVLHLEGEVNKIKSALLSTNKAVVSLSNGVSVLTFKVLDLKNYI

DKQLLPILNKQSCSISNIETVIEFQQKNNRLLEITREFSVNAGVTTPVSTYMLTN

SELLSLINDMPITNDQKKLMSNNVQIVRQQSYSIMCIIKEEVLAYVVQLPLYG

VIDTPCWKLHTSPLCTTNTKEGSNICLTRTDRGWYCDNAGSVSFFPQAETCK

VQSNRVFCDTMNSRTLPSEVNLCNVDIFNPKYDCKIMTSKTDVSSSVITSLGAI

VSCYGKTKCTASNKNRGIIKTFSNGCDYVSNKGVDTVSVGNTLYCVNKQEG

KSLYVKGEPIINFYDPLVFPSDEFDASISQVNEKINQSLAFIRKSDELLHNVNAG

KSTTNIMITTIIIVIIVILLSLIAVGLLLY

**The open reading frame (ORF) nucleic acid sequence of the MOF mRNA**

AUGGAGCUCUUGAUUCUGAAGGCCAACGCCAUCACCACCAUCCUGACAG

CCGUGACCUUCUGCUUCGCCAGCGGCCAGAACAUCACAGAGGAGUUCUA

CCAGAGCACCUGCUCCGCCGUGAGCAAGGGCUACCUGUCCGCCCUGCGG

ACAGGCUGGUACACCUCUGUGAUCACCAUCGAGCUGUCCAACAUCAAGG

AGAACAAGUGCAACGGCACCGACGCCAAGGUGAAGCUGAUCAAGCAGG

AGCUGGACAAGUACAAGAACGCCGUGACAGAGCUGCAGCUGCUGAUGC

AGAGCACCCCUGCCACCGGCAGCGGCAGCGCCAUCUGCAGCGGCGUGGC

CGUGUGCAAGGUGCUGCACCUGGAGGGCGAGGUGAACAAGAUCAAGAG

CGCCCUGCUGAGCACCAACAAGGCCGUGGUGUCUCUGAGCAACGGCGUG

AGCGUGCUGACCUUCAAGGUGCUGGACCUGAAGAACUACAUCGACAAG

CAGCUGCUGCCCAUCCUGAACAAGCAGAGCUGCUCCAUCUCCAACAUUG

AGACCGUGAUCGAGUUCCAGCAGAAGAACAACCGGCUGCUGGAGAUCA

CCCGGGAGUUCAGCGUGAACGCCGGGGUGACCACCCCCGUGAGCACCUA

CAUGCUGACCAACAGCGAGCUGCUGUCUCUGAUCAACGACAUGCCCAUC

ACCAACGACCAGAAGAAGCUGAUGAGCAACAACGUGCAGAUCGUGAGA

CAGCAGAGCUACUCCAUCAUGUGCAUCAUCAAGGAGGAGGUGCUGGCC

UACGUGGUGCAGCUGCCCCUGUAUGGGGUGAUCGACACCCCAUGCUGG

AAGCUGCACACCAGUCCACUGUGCACCACCAACACCAAGGAGGGCAGCA

ACAUCUGUCUGACCCGGACAGACCGGGGCUGGUAUUGCGACAAUGCCG

GCUCCGUGAGCUUCUUCCCUCAGGCCGAGACCUGCAAGGUGCAGAGCAA

CCGGGUGUUCUGCGACACCAUGAACUCUCGGACCCUGCCCUCCGAGGUG

AACCUGUGCAAUGUGGACAUCUUCAAUCCCAAGUACGAUUGCAAGAUC

AUGACCAGCAAGACCGAUGUGUCCAGCAGCGUGAUCACCAGCCUGGGG

GCCAUCGUGUCUUGCUACGGCAAGACAAAGUGCACCGCCUCCAACAAGA

ACAGGGGCAUCAUCAAGACCUUCUCCAACGGAUGCGACUACGUGUCCAA

CAAGGGCGUGGACACCGUGUCCGUGGGGAACACCCUGUACUGCGUGAA

CAAGCAGGAGGGGAAGAGCCUGUACGUGAAGGGCGAGCCCAUCAUCAA

CUUUUACGACCCCCUGGUGUUCCCCUCCGACGAGUUCGACGCCAGCAUC

AGCCAGGUGAAUGAGAAGAUCAAUCAGAGCCUGGCCUUCAUCAGAAAG

UCUGAUGAGCUGCUGCACAACGUGAACGCCGGCAAGAGCACCACCAACA

UCAUGAUCACCACCAUCAUCAUCGUGAUCAUCGUGAUCCUGCUGAGCCU

GAUCGCCGUGGGCCUGCUGCUGUACUAA

**The corresponding amino acid sequence of the MOF mRNA**

MELLILKANAITTILTAVTFCFASGQNITEEFYQSTCSAVSKGYLSALRTGWYT

SVITIELSNIKENKCNGTDAKVKLIKQELDKYKNAVTELQLLMQSTPATGSGS

AICSGVAVCKVLHLEGEVNKIKSALLSTNKAVVSLSNGVSVLTFKVLDLKNYI

DKQLLPILNKQSCSISNIETVIEFQQKNNRLLEITREFSVNAGVTTPVSTYMLTN

SELLSLINDMPITNDQKKLMSNNVQIVRQQSYSIMCIIKEEVLAYVVQLPLYG

VIDTPCWKLHTSPLCTTNTKEGSNICLTRTDRGWYCDNAGSVSFFPQAETCK

VQSNRVFCDTMNSRTLPSEVNLCNVDIFNPKYDCKIMTSKTDVSSSVITSLGAI

VSCYGKTKCTASNKNRGIIKTFSNGCDYVSNKGVDTVSVGNTLYCVNKQEG

KSLYVKGEPIINFYDPLVFPSDEFDASISQVNEKINQSLAFIRKSDELLHNVNAG

KSTTNIMITTIIIVIIVILLSLIAVGLLLY

**Comparison of the MF and MOF mRNA sequences**

```
Score    Expect   Identities   GapsStrand
2156 bits(1167)  0.0 1411/1533(92%)   0/1533(0%)   Plus/Plus
MF   1      ATGGAGCTGCTGATCCTGAAGGCCAACGCCATCACGACCATCCTGACCGCCGTGACCTTC  60
            ||||||||  |||| |||||||||||||||||||| ||||||||||| ||||||||||||
MOF  1      ATGGAGCTCTTGATTCTGAAGGCCAACGCCATCACCACCATCCTGACAGCCGTGACCTTC  60

MF   61     TGCTTCGCCAGCGGGCAGAACATCACCGAGGAGTTCTACCAGTCCACCTGCTCCGCCGTG  120
            |||||||||||||| ||||||||||| |||||||||||||||  ||||||||||||||||
MOF  61     TGCTTCGCCAGCGGCCAGAACATCACAGAGGAGTTCTACCAGAGCACCTGCTCCGCCGTG  120
```

```
MF    121   AGCAAGGGCTACCTGTCTGCCCTGAGAACCGGCTGGTACACCAGCGTGATCACCATCGAG   180
            ||||||||||||||||| |||||| | || ||||||||||||   |||||||||||||||
MOF   121   AGCAAGGGCTACCTGTCCGCCCTGCGGACAGGCTGGTACACCTCTGTGATCACCATCGAG   180

MF    181   CTGTCCAACATCAAGGAGAACAAGTGCAACGGCACCGACGCCAAGGTGAAGCTGATCAAG   240
            ||||||||||||||||||||||||||||||||||||||||||||||||||||||||||||
MOF   181   CTGTCCAACATCAAGGAGAACAAGTGCAACGGCACCGACGCCAAGGTGAAGCTGATCAAG   240

MF    241   CAGGAGCTGGACAAGTACAAGAACGCAGTGACCGAGCTGCAGCTGCTGATGCAGAGCACA   300
            |||||||||||||||||||||||||| ||||| |||||||||||||||||||||||||||
MOF   241   CAGGAGCTGGACAAGTACAAGAACGCCGTGACAGAGCTGCAGCTGCTGATGCAGAGCACC   300

MF    301   CCAGCCACCGGTAGCGGGTCCGCCATTTGCTCCGGCGTGGCCGTGTGCAAGGTGCTGCAC   360
            || |||||||| |||||   |||||| |||  ||||||||||||||||||||||||||||
MOF   301   CCTGCCACCGGCAGCGGCAGCGCCATCTGCAGCGGCGTGGCCGTGTGCAAGGTGCTGCAC   360

MF    361   CTGGAGGGCGAGGTGAACAAGATCAAGAGCGCCCTGCTCTCCACCAACAAGGCCGTGGTG   420
            ||||||||||||||||||||||||||||||||||||||   |||||||||||||||||||
MOF   361   CTGGAGGGCGAGGTGAACAAGATCAAGAGCGCCCTGCTGAGCACCAACAAGGCCGTGGTG   420

MF    421   AGCCTGAGCAACGGGGTGAGCGTGCTGACCTTCAAGGTGCTGGACCTGAAGAACTACATC   480
               ||||||||||| |||||||||||||||||||||||||||||||||||||||||||||
MOF   421   TCTCTGAGCAACGGCGTGAGCGTGCTGACCTTCAAGGTGCTGGACCTGAAGAACTACATC   480

MF    481   GACAAGCAGCTGCTGCCTATCCTGAACAAGCAGAGCTGCAGCATCAGCAACATCGAGACC   540
            ||||||||||||||||| |||||||||||||||||||||  ||||  |||||| ||||||
MOF   481   GACAAGCAGCTGCTGCCCATCCTGAACAAGCAGAGCTGCTCCATCTCCAACATTGAGACC   540

MF    541   GTGATCGAGTTCCAGCAGAAGAACAACCGGCTGCTGGAGATCACCAGGGAGTTCAGCGTG   600
            |||||||||||||||||||||||||||||||||||||||||||||| |||||||||||||
MOF   541   GTGATCGAGTTCCAGCAGAAGAACAACCGGCTGCTGGAGATCACCCGGGAGTTCAGCGTG   600

MF    601   AACGCAGGGGTGACCACACCCGTGTCCACCTACATGCTGACCAACTCCGAGCTGCTGAGC   660
            ||||| ||||||||||| ||||||  |||||||||||||||||||  ||||||||||
MOF   601   AACGCCGGGGTGACCACCCCCGTGAGCACCTACATGCTGACCAACAGCGAGCTGCTGTCT   660

MF    661   CTGATCAACGATATGCCCATCACCAACGACCAGAAGAAGCTGATGAGCAACAACGTGCAG   720
            ||||||||||| ||||||||||||||||||||||||||||||||||||||||||||||||
MOF   661   CTGATCAACGACATGCCCATCACCAACGACCAGAAGAAGCTGATGAGCAACAACGTGCAG   720

MF    721   ATCGTGCGGCAGCAGTCCTACTCCATCATGTGCATCATCAAGGAGGAGGTGCTGGCCTAC   780
            |||||| | ||||||  |||||||||||||||||||||||||||||||||||||||||||
MOF   721   ATCGTGAGACAGCAGAGCTACTCCATCATGTGCATCATCAAGGAGGAGGTGCTGGCCTAC   780

MF    781   GTGGTGCAGCTGCCCCTGTACGGCGTGATCGACACCCCTTGCTGGAAGCTGCACACCAGC   840
            |||||||||||||||||||| || |||||||||||||| ||||||||||||||||||||
MOF   781   GTGGTGCAGCTGCCCCTGTATGGGGTGATCGACACCCCATGCTGGAAGCTGCACACCAGT   840

MF    841   CCTCTGTGCACCACCAACACGAAGGAGGGCAGCAATATCTGCCTGACCCGGACCGACAGG   900
            || ||||||||||||||||| |||||||||||||| ||||| ||||||||||| ||| ||
MOF   841   CCACTGTGCACCACCAACACCAAGGAGGGCAGCAACATCTGTCTGACCCGGACAGACCGG   900

MF    901   GGCTGGTACTGCGACAACGCCGGCAGCGTGTCCTTCTTTCCCCAGGCCGAGACCTGCAAG   960
```

```
            |||||||| |||||||| ||||||  ||||  |||||| || ||||||||||||||||||
MOF   901   GGCTGGTATTGCGACAATGCCGGCTCCGTGAGCTTCTTCCCTCAGGCCGAGACCTGCAAG   960

MF    961   GTGCAGTCCAACAGGGTGTTCTGCGACACCATGAACTCTCGCACCCTGCCCAGCGAGGTG   1020
            ||||||  |||| |||||||||||||||||||||||||||| |||||||||  |||||||
MOF   961   GTGCAGAGCAACCGGGTGTTCTGCGACACCATGAACTCTCGGACCCTGCCCTCCGAGGTG   1020

MF    1021  AACCTGTGCAACGTGGACATCTTCAACCCCAAGTACGACTGCAAGATCATGACCTCCAAG   1080
            ||||||||||| |||||||||||||| ||||||||||| |||||||||||||||  ||||
MOF   1021  AACCTGTGCAATGTGGACATCTTCAATCCCAAGTACGATTGCAAGATCATGACCAGCAAG   1080

MF    1081  ACCGACGTGTCCTCTAGCGTTATCACCTCCCTGGGCGCCATCGTGAGCTGCTACGGCAAG   1140
            ||||| ||||||   ||||| ||||||  |||||| |||||||||   ||||||||||||
MOF   1081  ACCGATGTGTCCAGCAGCGTGATCACCAGCCTGGGGGCCATCGTGTCTTGCTACGGCAAG   1140

MF    1141  ACCAAGTGCACCGCCAGCAACAAGAACAGGGGCATCATCAAGACCTTCAGCAACGGGTGC   1200
            || ||||||||||||  |||||||||||||||||||||||||||||||  |||||| |||
MOF   1141  ACAAAGTGCACCGCCTCCAACAAGAACAGGGGCATCATCAAGACCTTCTCCAACGGATGC   1200

MF    1201  GACTACGTGTCCAACAAGGGCGTGGACACCGTGTCCGTGGGCAACACCCTGTACTGCGTG   1260
            ||||||||||||||||||||||||||||||||||||||||| ||||||||||||||||||
MOF   1201  GACTACGTGTCCAACAAGGGCGTGGACACCGTGTCCGTGGGGAACACCCTGTACTGCGTG   1260

MF    1261  AACAAGCAGGAGGGCAAGAGCCTGTACGTGAAGGGCGAGCCCATCATCAACTTCTACGAC   1320
            |||||||||||||| |||||||||||||||||||||||||||||||||||||| ||||||
MOF   1261  AACAAGCAGGAGGGGAAGAGCCTGTACGTGAAGGGCGAGCCCATCATCAACTTTTACGAC   1320

MF    1321  CCTCTGGTGTTCCCCAGCGACGAGTTCGACGCCAGCATCTCCCAGGTGAACGAGAAGATC   1380
            || ||||||||||||  ||||||||||||||||||||||  ||||||||| |||||||||
MOF   1321  CCCCTGGTGTTCCCCTCCGACGAGTTCGACGCCAGCATCAGCCAGGTGAATGAGAAGATC   1380

MF    1381  AACCAGAGCCTGGCCTTCATCCGCAAGAGCGACGAGCTGCTGCACAACGTGAACGCCGGC   1440
            || |||||||||||||||||| | |||   || |||||||||||||||||||||||||||
MOF   1381  AATCAGAGCCTGGCCTTCATCAGAAAGTCTGATGAGCTGCTGCACAACGTGAACGCCGGC   1440

MF    1441  AAGAGCACCACAAACATCATGATCACCACCATCATCATCGTGATAATCGTGATCCTGCTG   1500
            ||||||||||| |||||||||||||||||||||||||||||||||| |||||||||||||||
MOF   1441  AAGAGCACCACCAACATCATGATCACCACCATCATCATCGTGATCATCGTGATCCTGCTG   1500

MF    1501  TCCCTGATCGCTGTGGGCCTGCTGCTGTACTAA   1533
              ||||||||| |||||||||||||||||||||
MOF   1501  AGCCTGATCGCCGTGGGCCTGCTGCTGTACTAA   1533
```
